# Comparative effectiveness of BNT162b2 versus mRNA-1273 covid-19 vaccine boosting in England: matched cohort study in OpenSAFELY-TPP

**DOI:** 10.1136/bmj-2022-072808

**Published:** 2023-03-15

**Authors:** William J Hulme, Elsie M F Horne, Edward P K Parker, Ruth H Keogh, Elizabeth J Williamson, Venexia Walker, Tom M Palmer, Helen J Curtis, Alex J Walker, Colm D Andrews, Amir Mehrkar, Jessica Morley, Brian MacKenna, Sebastian C J Bacon, Ben Goldacre, Miguel A Hernán, Jonathan A C Sterne

**Affiliations:** 1 The Bennett Institute for Applied Data Science, Nuffield Department of Primary Care Health Sciences, University of Oxford, Oxford, UK; 2 Population Health Sciences, University of Bristol, Bristol, UK; 3 NIHR Bristol Biomedical Research Centre, Bristol, UK; 4 London School of Hygiene and Tropical Medicine, London, UK; 5 MRC Integrative Epidemiology Unit, Bristol Medical School, University of Bristol, Bristol, UK; 6 CAUSALab, Harvard T.H. Chan School of Public Health, Boston, MA, USA; 7 Departments of Epidemiology and Biostatistics, Harvard T.H. Chan School of Public Health, Boston, MA, USA; 8 Health Data Research UK South West, Bristol, UK

## Abstract

**Objective:**

To compare the effectiveness of the BNT162b2 mRNA (Pfizer-BioNTech) and mRNA-1273 (Moderna) covid-19 vaccines during the booster programme in England.

**Design:**

Matched cohort study, emulating a comparative effectiveness trial.

**Setting:**

Linked primary care, hospital, and covid-19 surveillance records available within the OpenSAFELY-TPP research platform, covering a period when the SARS-CoV-2 delta and omicron variants were dominant.

**Participants:**

3 237 918 adults who received a booster dose of either vaccine between 29 October 2021 and 25 February 2022 as part of the national booster programme in England and who received a primary course of BNT162b2 or ChAdOx1.

**Intervention:**

Vaccination with either BNT162b2 or mRNA-1273 as a booster vaccine dose.

**Main outcome measures:**

Recorded SARS-CoV-2 positive test, covid-19 related hospital admission, covid-19 related death, and non-covid-19 related death at 20 weeks after receipt of the booster dose.

**Results:**

1 618 959 people were matched in each vaccine group, contributing a total 64 546 391 person weeks of follow-up. The 20 week risks per 1000 for a positive SARS-CoV-2 test were 164.2 (95% confidence interval 163.3 to 165.1) for BNT162b2 and 159.9 (159.0 to 160.8) for mRNA-1273; the hazard ratio comparing mRNA-1273 with BNT162b2 was 0.95 (95% confidence interval 0.95 to 0.96). The 20 week risks per 1000 for hospital admission with covid-19 were 0.75 (0.71 to 0.79) for BNT162b2 and 0.65 (0.61 to 0.69) for mRNA-1273; the hazard ratio was 0.89 (0.82 to 0.95). Covid-19 related deaths were rare: the 20 week risks per 1000 were 0.028 (0.021 to 0.037) for BNT162b2 and 0.024 (0.018 to 0.033) for mRNA-1273; hazard ratio 0.83 (0.58 to 1.19). Comparative effectiveness was generally similar within subgroups defined by the primary course vaccine brand, age, previous SARS-CoV-2 infection, and clinical vulnerability. Relative benefit was similar when vaccines were compared separately in the delta and omicron variant eras.

**Conclusions:**

This matched observational study of adults estimated a modest benefit of booster vaccination with mRNA-1273 compared with BNT162b2 in preventing positive SARS-CoV-2 tests and hospital admission with covid-19 20 weeks after vaccination, during a period of delta followed by omicron variant dominance.

## Introduction

The UK covid-19 vaccination programme delivered its first “booster” doses in September 2021.^1^ Based on guidance from the Joint Committee for Vaccination and Immunisation (JCVI),^2^ booster vaccination was initially offered to groups at high risk of severe covid-19 and was then progressively extended to the whole adult population by mid-December 2021.^3^ ^4^ ^5^ The BNT162b2 Pfizer-BioNTech vaccine was used initially, with a half dose of mRNA-1273 Moderna vaccine also used from 29 October 2021 onwards. Concurrent boosting with BNT162b2 and mRNA-1273, receipt of which was largely determined by local availability rather than clinical criteria, enables a direct comparison of their effectiveness against positive SARS-CoV-2 tests and severe covid-19. No randomised trials have made such a comparison.

On behalf of NHS England, we used the OpenSAFELY-TPP database, covering 40% of English primary care practices and linked to national coronavirus surveillance, hospital episodes, and death registry data, to compare the effectiveness of boosting with BNT162b2 and mRNA-1273 in adults. Follow-up encompassed 29 October 2021 until 1 July 2022, a period of delta then omicron variant dominance.

## Methods

### Data sources

All data were linked, stored, and analysed securely within the OpenSAFELY platform (https://opensafely.org/). With the approval of NHS England, primary care records managed by the general practice software provider TPP were linked, using NHS numbers, to emergency department attendance and inpatient hospital spell records via NHS Digital’s Hospital Episode Statistics, national coronavirus testing records via the Second Generation Surveillance System (SGSS), and national death registry records from the Office for National Statistics. Covid-19 vaccination history and health and social care worker status are available in the general practice record directly via the National Immunisation Management System. The availability of free polymerase chain reaction (PCR) and lateral flow tests for SARS-CoV-2 in England ended on 31 March 2022, so we did not use testing data after this date.

### Vaccination strategies

The two strategies of interest were a third vaccine dose of BNT162b2 or mRNA-1273. We did not attempt to distinguish between third primary doses and first booster doses, as this information is not unambiguously ascertainable in the health record. However, most recipients of a third primary dose will have had their third dose before the study period.

### Eligibility criteria

People who received a third dose of BNT162b2 or mRNA-1273 between 29 October 2021 and 25 February 2022 inclusive, during which time both vaccine brands were being used, were considered for inclusion and classified in the corresponding group. Third dose recipients were eligible if they were aged 18 years and over; were registered at a general practice using TPP’s SystmOne clinical information system at the time of boosting; received a two dose primary vaccination course of either BNT162b2 or ChAdOx1-S (we did not consider mixed dosing and mRNA-1273 owing to small numbers); were not a health or social care worker, not resident in a care or nursing home, and not medically housebound or receiving end-of-life care; had no evidence of SARS-CoV-2 infection or covid-19 within the previous 28 days; were not admitted to hospital at the time of boosting; and had complete information on sex, deprivation, and Sustainability and Transformation Partnership (STP, a geographical grouping of NHS and local authorities).

### Matching

**Fig 1:**
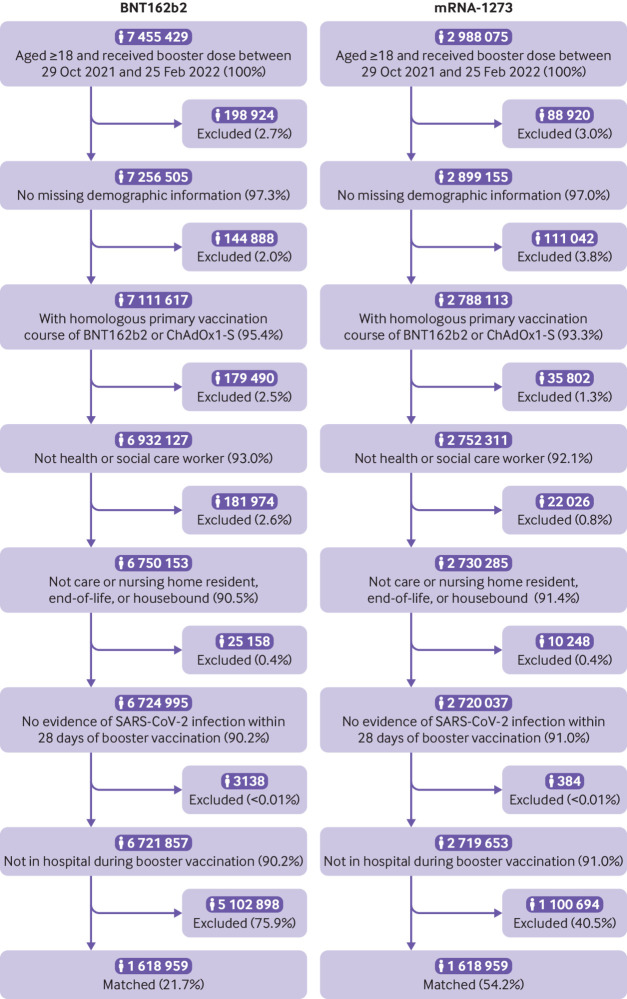
Flow of participants into study

We matched BNT162b2 and mRNA-1273 recipients one to one without replacement on the following characteristics: date of third dose (exactly), primary vaccine course (BNT162b2 or ChadOx1-S), date of second vaccine dose (seven day caliper), sex (male or female), age (three year caliper and within age groups defined by JVCI risk groups), clinical risk group defined by JCVI (clinically extremely vulnerable, clinically at risk, neither); Index of Multiple Deprivation (grouped by fifths), STP as a surrogate for geographical region, evidence of previous SARS-CoV-2 infection (any of positive SARS-CoV-2 test, probable infection documented in primary care, or covid-19 related hospital attendance or admission), and morbidity count (grouped as no, one, or two or more conditions from the following list: diabetes, body mass index >40, chronic heart disease, chronic kidney disease, chronic liver disease, chronic respiratory disease or severe asthma, chronic neurological disease, cancer within three years). No values were missing for these variables; we excluded people with missing values for age, sex, Index of Multiple Deprivation, or STP (2.8% of all adults receiving a third dose during the study period; see figure 1), and we defined all other variables by the presence or absence of clinical codes or events. The supplementary materials provide more information on how these characteristics were chosen and defined.

### Outcomes and follow-up

We considered three covid-19 related outcomes. We identified positive SARS-CoV-2 tests by using SGSS testing records and on the basis of swab date. We used the first positive test after baseline, regardless of whether this was the first ever positive test for each person. We included both PCR and lateral flow test results, without differentiating between symptomatic and asymptomatic infection. As both PCR tests and lateral flow tests are highly specific, we do not consider negative tests around the same time to undermine the conclusiveness of a positive test. We identified covid-19 related hospital admission by using Secondary Use Service inpatient hospital records with ICD-10 (international statistical classification of diseases, 10th revision) codes U07.1, U07.2, or U.909 as the primary or non-primary reason for admission. We defined covid-19 related death as death with U07.1, U07.2, or U.909 ICD-10 codes mentioned anywhere on the death certificate (that is, as an underlying or contributing cause of death). We also report on non-covid-19 related deaths.

We followed each person from receipt of a third vaccine dose (time zero) until the outcome of interest, with censoring at death, practice de-registration, 28 weeks, or the follow-up end date, which was 31 March 2022 for positive SARS-CoV-2 tests (coinciding with the end of freely available community testing in England) and 1 July 2022 otherwise.

### Statistical analysis

We tabulated baseline characteristics of each vaccine group and examined between group balance by using standardised mean differences. We estimated the cumulative incidence of each outcome in each vaccine group by using the Kaplan-Meier estimator. We estimated cumulative risk differences and risk ratios comparing the vaccine groups for each outcome. These were estimated up to 28 weeks where available, but we report estimates at 20 weeks as the primary comparison owing to the truncated follow-up for SARS-CoV-2 tests. We also used Cox models to estimate hazard ratios over the first 20 weeks and within period specific intervals defined by splits on weeks 1, 2, 4, 8, 12, 16, 20, 24, and 28, where available.

We used standard errors on the log-Kaplan-Meier scale to derive 95% confidence limits for the cumulative incidences. We derived confidence limits for the risk differences from the sum of squares of the standard errors on the Kaplan-Meier scale, using Greenwood’s formula. We derived confidence limits for the risk ratio from the sum of squares of the standard errors on the log-Kaplan-Meier scale.

#### Subgroup and secondary analyses

We estimated comparative effectiveness separately in the following subgroups: primary vaccine course (ChAdOx1-S or BNT162b2); age band (18-39, 40-54, 55-64, 65-74, ≥75 years); clinical vulnerability, as defined by the JCVI (not clinically at risk, clinically at risk, or clinically extremely vulnerable); and evidence of previous SARS-CoV-2 infection or not. We used χ^2^ tests to examine evidence for heterogeneity in comparative effectiveness between subgroups. We reported the rates of SARS-CoV-2 tests undertaken from baseline up to 31 March 2022 in each vaccine group, as lower testing rates in one group might lead to under-ascertainment of outcomes, particularly positive SARS-CoV-2 tests. We reported outcomes at one week, during which time the vaccine is not expected to have any protective immunological effect, as a negative control outcome.^6^

In a post hoc analysis, we compared vaccine effectiveness separately in the delta and omicron SARS-CoV-2 variant eras, defined as before versus on or after 15 December 2021. Follow-up for the delta era analysis was censored at 14 December 2021, so maximum follow-up time in this analysis is only 40 days. The omicron era analysis included all matched pairs vaccinated on or after 15 December 2021 and pairs vaccinated earlier in which both members of the pair remained under follow-up on 15 December 2021.

#### Disclosure control

To satisfy strict re-identification minimisation requirements for statistical outputs from OpenSAFELY’s Trusted Research Environment, we rounded counts to the nearest three, nine, 15, and so on. We rounded plots of cumulative event counts and the Kaplan-Meier cumulative incidence estimates such that each increment is based on at least six events. Event rates, risk differences, and risk ratios were derived from these rounded estimates.

#### Software, code, and reproducibility

We used OpenSAFELY tools, Python version 3.8.10, and R version 4.0.2 for data management and analyses. All code is shared openly for review and re-use under MIT open licence at https://github.com/opensafely/comparative-booster. Codelists are referenced in supplementary materials and viewable at https://www.opencodelists.org/. The supplementary materials provide further details of definitions and data sources used for all variables in the study. Detailed pseudonymised patient data are potentially re-identifiable and therefore not shared.

### Patient and public involvement

Public contributors were not involved in setting the research question or the outcome measures, nor in developing plans for design or implementation of the study. No public contributors were asked to advise on interpretation or writing up of results. Covid-19 vaccination is offered to the whole population, including the researchers involved in the study.

## Results

### Study population and matching

A total of 10 443 504 adults registered at a TPP practice received a third vaccine dose of either BNT162b2 (7 455 429) or mRNA-1273 (2 988 075) during the study period, with 6 721 857 (90.2%) and 2 719 653 (91.0%) eligible for matching (fig 1). Eligible BNT162b2 third dose recipients were on average older and more deprived and had higher rates of previous clinical conditions than mRNA-1273 third dose recipients (supplementary table S1). They were also more likely to have received BNT162b2 as their primary vaccination course (38% *v* 30%).

After matching, 1 618 959 people in each vaccine group (total 3 237 918) were included in analyses, representing 24.1% and 59.5% of eligible BNT162b2 and mRNA-1273 recipients respectively (fig 1 and supplementary figure S1). Characteristics at the start of follow-up were well balanced between groups (table 1), with standardised mean differences consistently below 0.05 (supplementary figure S2 and table S1). In particular, previous clinical conditions, which were not directly matched on, were well balanced between the groups after, although not before, matching. The total follow-up time across both groups was 64 546 391 person weeks. The median follow-up was 15.3 weeks for positive SARS-CoV-2 tests and 28.0 weeks for other outcomes (supplementary table S3). SARS-CoV-2 testing rates after baseline were slightly less frequent for BNT162b2 than for mRNA-1273 (0.139 *v* 0.151 per week respectively; supplementary table S4).

**Table 1:** Baseline characteristics before and after matching. Values are numbers (percentages) unless stated otherwise

Variable	BNT162b2 (n=1 618 959)	mRNA-1273 (n=1 618 959)	Standardised mean difference
Primary vaccine course:			
BNT162b2-BNT162b2	476 211 (29.4)	476 211 (29.4)	-
ChAdOx1-ChAdOx1	1 142 745 (70.6)	1 142 745 (70.6)	-
Mean (SD) days between dose 2 and 3*	179.3 (26.9)	179.2 (26.8)	−0.005
Mean (SD) age, years	49.3 (13.8)	49.3 (13.8)	0.000
Age band:			
18-39	448 437 (27.7)	448 437 (27.7)	-
40-49	344 865 (21.3)	344 865 (21.3)	-
50-54	238 179 (14.7)	238 179 (14.7)	-
55-59	235 779 (14.6)	235 779 (14.6)	-
60-64	183 423 (11.3)	183 423 (11.3)	-
65-69	102 165 (6.3)	102 165 (6.3)	-
70-74	49 305 (3.0)	49 305 (3.0)	-
75-79	13 011 (0.8)	13011 (0.8)	-
≥80	3789 (0.2)	3789 (0.2)	-
Female sex	770 097 (47.6)	770 097 (47.6)	-
Ethnicity:			
White	1 379 673 (85.2)	1 378 173 (85.1)	−0.003
Black	14 901 (0.9)	14 565 (0.9)	−0.002
South Asian	70 923 (4.4)	67 365 (4.2)	−0.011
Mixed	12 993 (0.8)	13 119 (0.8)	0.001
Other	24 375 (1.5)	24 939 (1.5)	0.003
Unknown	116 085 (7.2)	120 795 (7.5)	0.011
Deprivation:			
1 (most deprived)	196 185 (12.1)	196 185 (12.1)	-
2	277 887 (17.2)	277 887 (17.2)	-
3	371 733 (23.0)	371 733 (23.0)	-
4	388 203 (24.0)	388 203 (24.0)	-
5 (least deprived)	384 951 (23.8)	384 951 (23.8)	-
Clinical vulnerability:			
Not at risk	1 403 499 (86.7)	1 403 499 (86.7)	-
At risk	199 203 (12.3)	199 203 (12.3)	-
Extremely vulnerable	16 257 (1.0)	16 257 (1.0)	-
Body mass index >40	20 691 (1.3)	20 661 (1.3)	0.000
Chronic heart disease	73 101 (4.5)	73 977 (4.6)	0.003
Chronic kidney disease	20 493 (1.3)	20 223 (1.2)	−0.001
Diabetes	65 871 (4.1)	65 505 (4.0)	−0.001
Chronic liver disease	21 441 (1.3)	21 447 (1.3)	0.000
Chronic respiratory disease	20 667 (1.3)	20 373 (1.3)	−0.002
Asthma	3753 (0.2)	3783 (0.2)	0.000
Chronic neurological disease	36 243 (2.2)	35 841 (2.2)	−0.002
Morbidity count:			
0	1 399 287 (86.4)	1 399 287 (86.4)	-
1	177 387 (11.0)	177 387 (11.0)	-
≥2	42 285 (2.6)	42 285 (2.6)	-
Any immunosuppressive condition	24 915 (1.5)	24 435 (1.5)	−0.002
Asplenia or poor spleen function	2805 (0.2)	2817 (0.2)	0.000
Cancer (excluding haematological), within previous 3 years	15 621 (1.0)	15 741 (1.0)	0.001
Haematological cancer, within previous 3 years	1029 (0.1)	861 (0.1)	−0.004
Solid organ transplant	273 (0.0)	225 (0.0)	−0.002
Immunosuppressive drugs, within 6 months	2865 (0.2)	2391 (0.1)	−0.007
HIV/AIDS	15 (0.0)	21 (0.0)	0.001
Learning disabilities	2997 (0.2)	2157 (0.1)	−0.013
Serious mental illness	4869 (0.3)	4431 (0.3)	−0.005
No of SARS-CoV-2 tests:			
0	786 687 (48.6)	758 883 (46.9)	−0.034
1	313 875 (19.4)	313 647 (19.4)	0.000
2	158 841 (9.8)	162 681 (10.0)	0.008
≥3	359 547 (22.2)	383 745 (23.7)	0.036
Previous documented SARS-CoV-2 infection	149 685 (9.2)	149 685 (9.2)	-

Standardised mean differences reported as “-” indicate that variable was exactly matched on and is therefore zero by design.

SD=standard deviation.

### Estimated comparative effectiveness

**Fig 2:**
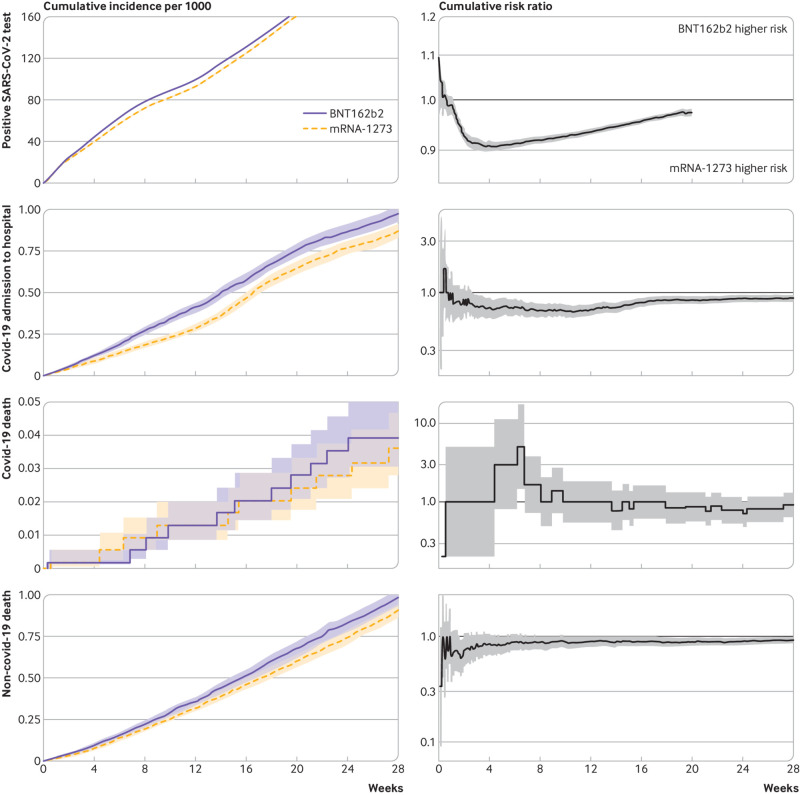
Estimates of cumulative risk per 1000 people (left plots) and cumulative risk ratios (right plots)

By 20 weeks, 412 038 positive SARS-CoV-2 tests, 2250 covid-19 related hospital admissions, and 84 covid-19 related deaths had occurred (table 2). The 20 week risks (cumulative incidence) per 1000 people of a positive SARS-CoV-2 test were 164.2 (95% confidence interval 163.3 to 165.1) for those receiving BNT162b2 and 159.9 (159.0 to 160.8) for mRNA-1273 (risk difference −4.25, 95% confidence interval −5.53 to −2.98) (table 2; fig 2). The corresponding 20 week risks per 1000 people for covid-19 related hospital admission were 0.75 (0.71 to 0.79) for BNT162b2 and 0.65 (0.61 to 0.69) for mRNA-1273 (risk difference −0.10, −0.16 to −0.046). The 20 week risks per 1000 people of covid-19 related death were 0.028 (0.021 to 0.037) for BNT162b2 and 0.024 (0.018 to 0.033) for mRNA-1273 (risk difference −0.004, −0.015 to 0.007). Corresponding risks of non-covid-19 related death were 0.67 (0.63 to 0.71) for BNT162b2 and 0.59 (0.56 to 0.63) for mRNA-1273 (risk difference −0.078, −0.13 to−0.023).

**Table 2:** Estimates at 20 week follow-up of risk per 1000 people in each vaccine group and corresponding risk differences, overall and within subgroups

Subgroup	BNT162b2				mRNA-1273			Risk difference (95% CI); reference=BNT162b2
	Weeks	Events	Risk (95% CI)		Weeks	Events	Risk (95% CI)	
**Positive SARS-CoV-2 test**								
All	23 962 892	210 567	164.2 (163.3 to 165.1)		24 074 138	201 471	159.9 (159.0 to 160.8)	−4.25 (−5.53 to−2.98)
Primary vaccine course:								
ChAdOx1-S	17 468 525	139 281	153.1 (152.0 to 154.1)		17 535 358	134 727	150.3 (149.3 to 151.4)	−2.73 (−4.19 to−1.27)
BNT162b2	6 494 309	71 289	191.1 (189.3 to 193.0)		6 538 744	66 741	182.4 (180.5 to 184.2)	−8.73 (−11.3 to−6.12)
Age band:								
18-39	5 304 605	70 131	258.5 (249.5 to 267.9)		5 321 630	65 091	250.3 (241.7 to 259.3)	−8.20 (−20.9 to 4.52)
40-54	7 883 465	82 587	203.8 (200.6 to 207.0)		7 939 547	80 193	201.3 (198.0 to 204.5)	−2.53 (−7.11 to 2.05)
55-64	7 039 101	44 985	135.0 (133.1 to 136.9)		7 092 697	43 689	132.2 (130.3 to 134.1)	−2.84 (−5.52 to−0.17)
65-74	3 254 068	11 859	77.1 (75.7 to 78.5)		3 234 526	11 571	76.3 (74.9 to 77.8)	−0.77 (−2.79 to 1.25)
≥75	481 566	999	42.9 (40.3 to 45.7)		485 658	927	40.2 (37.7 to 42.9)	−2.71 (−6.42 to 1.00)
Previous SARS-CoV-2 infection:								
No	21 803 589	199 953	169.8 (168.9 to 170.8)		21 906 769	191 571	165.5 (164.5 to 166.4)	−4.39 (−5.71 to−3.08)
Yes	2 159 246	10 617	96.5 (92.8 to 100.4)		2 167 320	9897	91.7 (88.5 to 95.1)	−4.82 (−9.86 to 0.23)
Clinical vulnerability:								
Not at risk	20 337 399	188 967	173.3 (172.2 to 174.4)		20 438 521	180 513	168.8 (167.7 to 169.9)	−4.48 (−6.07 to−2.90)
At risk	3 345 402	19 947	118.4 (116.6 to 120.1)		3 353 736	19 431	116.3 (114.6 to 118.1)	−2.06 (−4.52 to 0.41)
Extreme	280 104	1659	116.8 (111.3 to 122.5)		281 904	1527	108.4 (103.0 to 114.0)	−8.39 (−16.2 to−0.54)
**Covid-19 related hospital admission**								
All	32 271 647	1209	0.75 (0.71 to 0.79)		32 255 892	1041	0.65 (0.61 to 0.69)	−0.10 (−0.16 to−0.046)
Primary vaccine course:								
ChAdOx1-S	22 799 965	885	0.78 (0.73 to 0.83)		22 795 404	771	0.68 (0.63 to 0.73)	−0.100 (−0.17 to−0.030)
BNT162b2	9 471 640	321	0.68 (0.61 to 0.76)		9 460 434	267	0.56 (0.50 to 0.64)	−0.11 (−0.21 to−0.012)
Age band:								
18-39	8 292 039	165	0.40 (0.34 to 0.46)		8 237 363	141	0.34 (0.29 to 0.40)	−0.055 (−0.14 to 0.028)
40-54	11 038 062	285	0.52 (0.46 to 0.58)		11 061 422	249	0.45 (0.40 to 0.51)	−0.066 (−0.15 to 0.016)
55-64	8 818 450	351	0.80 (0.72 to 0.88)		8 860 096	255	0.58 (0.51 to 0.65)	−0.22 (−0.33 to−0.11)
65-74	3 612 425	267	1.48 (1.31 to 1.67)		3 582 778	255	1.42 (1.26 to 1.61)	−0.054 (−0.30 to 0.19)
≥75	510 603	141	5.52 (4.68 to 6.50)		514 155	135	5.25 (4.43 to 6.21)	−0.27 (−1.54 to 1.00)
Previous SARS-CoV-2 infection:								
No	29 289 145	1155	0.79 (0.74 to 0.84)		29 274 029	1005	0.69 (0.65 to 0.73)	−0.10 (−0.16 to−0.040)
Yes	2 982 451	57	0.38 (0.29 to 0.50)		2 981 813	33	0.22 (0.16 to 0.31)	−0.16 (−0.29 to−0.036)
Clinical vulnerability:								
Not at risk	27 979 286	657	0.47 (0.44 to 0.51)		27 962 914	567	0.41 (0.37 to 0.44)	−0.064 (−0.11 to−0.015)
At risk	3 970 272	447	2.25 (2.05 to 2.47)		3 970 212	369	1.86 (1.68 to 2.06)	−0.39 (−0.67 to−0.11)
Extreme	322 123	111	6.88 (5.71 to 8.28)		322 793	99	6.13 (5.03 to 7.46)	−0.75 (−2.51 to 1.00)
**Covid-19 related death**								
All	32 282 267	45	0.028 (0.021 to 0.037)		32 264 124	39	0.024 (0.018 to 0.033)	−0.004 (−0.015 to 0.007)
Primary vaccine course:								
ChAdOx1-S	22 807 701	33	0.029 (0.021 to 0.041)		22 801 517	27	0.024 (0.016 to 0.035)	−0.005 (−0.019 to 0.008)
BNT162b2	9 474 520	15	0.032 (0.019 to 0.053)		9 462 563	9	0.019 (0.010 to 0.037)	−0.013 (−0.033 to 0.008)
Age band:								
18-39	8 293 904	0	0.000 (NA to NA)		8 238 648	3	0.007 (0.002 to 0.023)	0.007 (−0.001 to 0.016)
40-54	11 040 830	9	0.016 (0.008 to 0.031)		11 063 617	3	0.005 (0.002 to 0.017)	−0.011 (−0.023 to 0.001)
55-64	8 821 432	9	0.020 (0.011 to 0.039)		8 862 073	9	0.020 (0.011 to 0.039)	0.000 (−0.019 to 0.019)
65-74	3 614 392	9	0.050 (0.026 to 0.096)		3 584 586	9	0.050 (0.026 to 0.096)	0.000 (−0.046 to 0.047)
≥75	511 621	15	0.59 (0.35 to 0.97)		515 106	9	0.35 (0.18 to 0.67)	−0.24 (−0.61 to 0.14)
Previous SARS-CoV-2 infection:								
No	29 299 190	45	0.031 (0.023 to 0.041)		29 281 899	33	0.023 (0.016 to 0.032)	−0.008 (−0.020 to 0.004)
Yes	2 983 022	0	0.000 (NA to NA)		2 982 171	3	0.020 (0.006 to 0.062)	0.020 (−0.003 to 0.043)
Clinical vulnerability:								
Not at risk	27 985 404	15	0.011 (0.006 to 0.018)		27 967 680	15	0.011 (0.006 to 0.018)	0.000 (−0.008 to 0.008)
At risk	3 973 871	27	0.14 (0.093 to 0.20)		3 972 932	15	0.076 (0.046 to 0.13)	−0.060 (−0.12 to 0.003)
Extreme	323 013	3	0.18 (0.060 to 0.57)		323 532	3	0.18 (0.060 to 0.57)	0.000 (−0.30 to 0.30)
**Non-covid-19 related death**								
All	32 282 267	1083	0.67 (0.63 to 0.71)		32 264 124	957	0.59 (0.56 to 0.63)	−0.078 (−0.13 to−0.023)
Primary vaccine course:								
ChAdOx1-S	22 807 701	831	0.73 (0.68 to 0.78)		22 801 517	759	0.67 (0.62 to 0.71)	−0.063 (−0.13 to 0.006)
BNT162b2	9 474 520	249	0.53 (0.46 to 0.60)		9 462 563	195	0.41 (0.36 to 0.47)	−0.11 (−0.20 to−0.026)
Age band:								
18-39	8 293 904	27	0.065 (0.045 to 0.095)		8 238 648	27	0.066 (0.045 to 0.096)	0.000 (−0.035 to 0.035)
40-54	11 040 830	141	0.26 (0.22 to 0.30)		11 063 617	147	0.27 (0.23 to 0.31)	0.010 (−0.050 to 0.070)
55-64	8 821 432	321	0.73 (0.65 to 0.81)		8 862 073	279	0.63 (0.56 to 0.71)	−0.098 (−0.21 to 0.010)
65-74	3 614 392	375	2.07 (1.87 to 2.29)		3 584 586	345	1.92 (1.73 to 2.14)	−0.15 (−0.44 to 0.14)
≥75	511 621	219	8.53 (7.48 to 9.74)		515 106	159	6.16 (5.27 to 7.19)	−2.37 (−3.85 to−0.90)
Previous SARS-CoV-2 infection:								
No	29 299 190	1041	0.71 (0.67 to 0.76)		29 281 899	933	0.64 (0.60 to 0.68)	−0.073 (−0.13 to−0.014)
Yes	2 983 022	39	0.26 (0.19 to 0.36)		2 982 171	21	0.14 (0.092 to 0.22)	−0.12 (−0.22 to−0.019)
Clinical vulnerability:								
Not at risk	27 985 404	423	0.30 (0.27 to 0.33)		27 967 680	417	0.30 (0.27 to 0.33)	−0.004 (−0.045 to 0.037)
At risk	3 973 871	489	2.46 (2.25 to 2.69)		3 972 932	423	2.13 (1.93 to 2.34)	−0.33 (−0.63 to−0.034)
Extreme	323 013	165	10.2 (8.74 to 11.8)		323 532	111	6.84 (5.68 to 8.24)	−3.33 (−5.33 to−1.33)

Counts and survival estimates are based on values rounded up to nearest 6*n*–3 for disclosure control.

CI=confidence interval.

**Fig 3:**
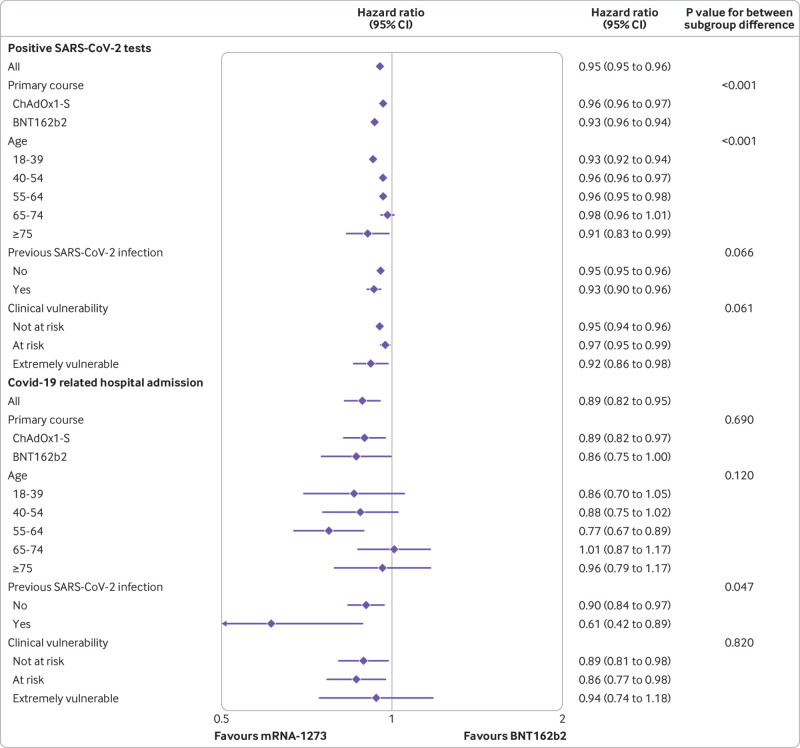
Estimated hazard ratios at 20 week follow-up comparing effectiveness of mRNA-1273 and BNT162b2 on SARS-CoV-2 infection and covid-19 related hospital admission, overall and within subgroups, together with P values for between subgroup heterogeneity. CI=confidence interval

**Fig 4:**
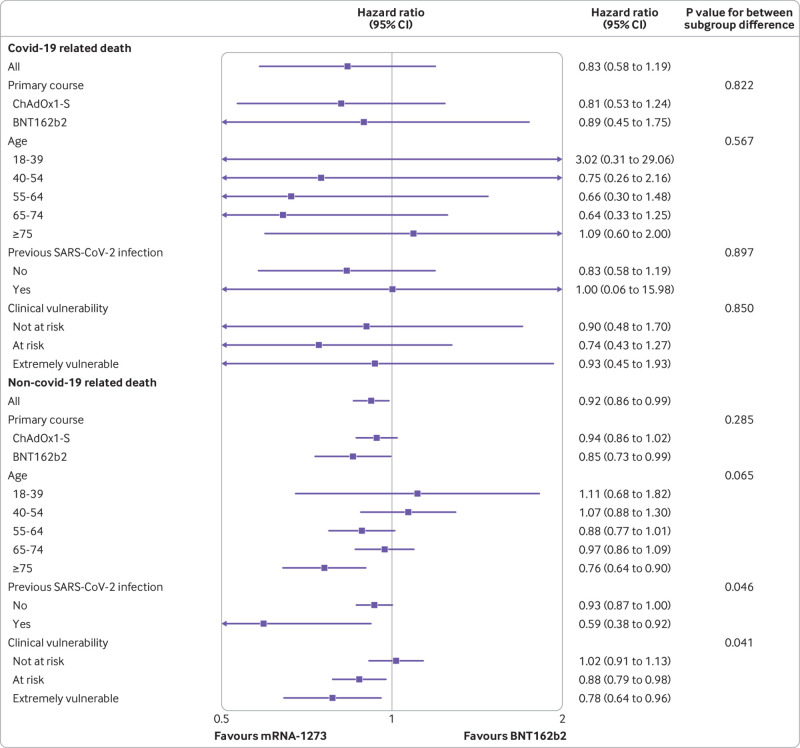
Estimated hazard ratios at 20 week follow-up comparing effectiveness of mRNA-1273 and BNT162b2 on covid-19 related and non-covid-19 related death, overall and within subgroups, together with P values for between subgroup heterogeneity. CI=confidence interval

For positive SAR-CoV-2 tests, the cumulative risk ratio comparing mRNA-1273 with BNT162b2 reached a minimum of 0.91 (95% confidence interval 0.90 to 0.92) at four weeks and then attenuated with increasing follow-up (fig 2). For covid-19 related hospital admission, the minimum risk ratio was 0.67 (0.59 to 0.76) at around 11 weeks, attenuating to 0.89 (0.83 to 0.96) by 28 weeks. The number of covid-19 related deaths was too small for us to be able to reliably examine patterns of cumulative incidence over time. For non-covid-19 related death, the risk ratio was imprecisely estimated in the first few weeks but stabilised to 0.92 (0.86 to 0.99) at 28 weeks. Hazard ratios (whereby <1 favours mRNA-1273) in the 20 week period after receipt of third dose were 0.95 (95% confidence ratio 0.95 to 0.96) for positive SARS-CoV-2 test, 0.89 (0.82 to 0.95) for covid-19 related hospital admission, 0.83 (0.58 to 1.19) for covid-19 related death, and 0.92 (0.86 to 0.99) for non-covid-19 related death (fig 3 and fig 4).

Period specific hazard ratios are shown in supplementary figure S4a. During the first week after vaccination, event rates were similar between vaccine groups: the estimated hazard ratio was 0.99 (0.97 to 1.01) for positive SARS-CoV-2 tests, 0.86 (0.54 to 1.39) for covid-19 related hospital admission, 1.00 (0.06 to 15.99) for covid-19 related death, and 0.92 (0.51 to 1.63) for non-covid-19 related death. After this initial period, the rate for positive SARS-CoV-2 tests was lower for mRNA-1273 than BNT162b2 until week 12, after which it was higher for mRNA-1273. For covid-19 related hospital admission, the minimum hazard ratio was 0.64 (0.53 to 0.79) during weeks 5-8, with hazard ratios between 12 and 28 weeks consistent with no difference between vaccine groups. For non-covid-19 related death, hazard ratios were close to 1 during weeks 3-28.

### Subgroup analyses

Absolute risks differed between subgroups, and therefore so did the absolute risk differences comparing vaccines, although mRNA-1273 was consistently associated with lower risk than BNT162b2 for positive SARS-CoV-2 tests and covid-19 related hospital admission (table 2).

For positive SARS-CoV-2 tests, the estimated relative benefit of mRNA-1273 compared with BNT162b2 was greater in people who received BNT162b2 for their first two vaccine doses (hazard ratio 0.93, 0.92 to 0.94) than in those who received ChAdOx1 (0.96, 0.96 to 0.97; P for heterogeneity<0.001) (fig 3). Similarly, the estimated relative benefit of mRNA-1273 compared with BNT162b2 was greater in people with previous evidence of infection (hazard ratio 0.93, 0.90 to 0.96) than in those without (0.95, 0.95 to 0.96; P for heterogeneity=0.066). For covid-19 related hospital admission, the estimated relative benefit of mRNA-1273 compared with BNT162b2 was similar by type of first two vaccine doses (hazard ratio 0.86 (0.75 to 1.00) for BNT162b2 and 0.89 (0.82 to 0.97) for ChAdOx1; P for heterogeneity=0.690). The estimated relative benefit of mRNA-1273 was greater in people with previous evidence of infection (hazard ratio 0.61, 0.42 to 0.89) than in those without (0.90, 0.84 to 0.97; P for heterogeneity=0.047). For covid-19 related death, hazard ratios were estimated imprecisely owing to low event rates, so no strong conclusions about variation between subgroups could be made. For non-covid-19 related death, the relative benefit of mRNA-1273 was generally greater in older and more clinically vulnerable people.

Supplementary materials provide cumulative incidence plots by subgroup (figures S3a-e); the corresponding cumulative risk differences, cumulative risk ratios, and period specific hazard ratios (figures S4a-e); and subgroup specific heterogeneity tests for risk differences and risk ratios (table S2).

### Variant era analyses

Hazard ratios estimated for follow-up time restricted to 14 December 2021 (delta era) and restricted to matched pairs who remained at risk on or who were vaccinated after 15 December 2021 (omicron era) were similar or else very imprecisely estimated (supplementary figure S5 and table S5).

## Discussion

This observational study in more than 3.2 million adults with a primary covid-19 vaccine dose of BNT162b2 only or ChAdOx1 only compared the effectiveness of mRNA-1273 versus BNT162b2 vaccines for a third dose and is, to our knowledge, the first to do so against severe covid-19 outcomes. We estimated that mRNA-1273 provided better protection than BNT162b2 against a positive SARS-CoV-2 test (hazard ratio 0.95, 0.95 to 0.96) and, particularly, covid-19 related hospital admission (0.89, 0.82 to 0.95) in the first 20 weeks after receipt of a third dose, although period specific hazard ratios suggest that this benefit is largely restricted to the first 10 weeks. Estimates seemed broadly similar regardless of age, clinical vulnerability, and whether evidence of previous infection existed.

The risk of a positive SARS-CoV-2 test within 20 weeks of the third dose was estimated at around 1 in 6 people, reflecting both the high infection rates in the study period, particularly from December 2021 onward as the omicron variant become dominant, and the modest protection against infection offered by a third vaccine dose. Rates of positive SARS-CoV-2 tests were higher (the cumulative incidence curve is steeper) during the immune induction period up to two weeks, during which time the vaccine has yet to take full effect, than subsequently. Consistent with this delayed immunity, rates of the outcomes were similar for the two vaccines in the first week of follow-up. After this period, an apparent modest benefit of mRNA-1273 over BNT162b2 emerged. Period specific hazard ratios suggest that this benefit was due to relatively higher protection between weeks 2 and 12, which reversed from week 16. Covid-19 related hospital admission was rare (around 1 event in 1450) within 20 weeks of vaccination. The hazard ratios in weeks 3 to 12 favoured mRNA-1273, although they were around 1 after week 12.

Only 84 people died with covid-19 recorded as an underlying or contributory cause across both vaccine groups within 20 weeks (1 in 38 500), so the hazard ratio was estimated imprecisely. The risk of non-covid-19 related death was around 1 in 1600 people and was marginally lower for mRNA-1273 than BNT162b2.

### Strengths and limitations of study

The OpenSAFELY-TPP database covers around 40% of the English population and contains rich clinical information that enabled us to closely match BNT162b2 and mRNA-1273 recipients to control for potential confounding, to study a range of clinical outcomes, including severe covid-19, and to compare comparative effectiveness between important clinical subgroups.

To make fair comparisons between the two vaccine types, we exploited the concurrent roll-out of both vaccines across the same eligible population in the same time period. The type of vaccine administered was based largely on local supply and availability, and clinical characteristics did not inform the type of vaccine offered on-site. Controlling for any potential differences in the distribution of prognostic factors between the two vaccine groups remained important, and these were well balanced after matching. We cannot rule out residual confounding, as we did not match on or adjust for all potential confounders—for example, some clinical subgroups in which relative protection may differ between vaccines, such as immunosuppressive conditions. However, unmeasured potential confounders, particularly relating to unmeasured health seeking behaviours, are less problematic in comparative effectiveness studies than in studies comparing vaccinated and unvaccinated people, as all people entering the study have sought and received at three doses of vaccine. Furthermore, rates of positive SARS-CoV-2 tests were similar during the first week after vaccination, suggesting that the vaccine groups were similar at the start of follow-up. We found no evidence that rates of other outcomes during the first week after vaccination differed between the vaccine groups, although some of these comparisons were estimated imprecisely.

We excluded groups who were unlikely to undergo boosting, such as those with a recent positive test or in palliative care. We also excluded people living in care homes and health and social care workers, as these groups are much more likely to receive their vaccines at their place of residence or work rather than at walk-in or bookable community vaccination centres, which may introduce bias. For example, occupation type for healthcare workers may influence both setting of vaccine administration (and therefore vaccine type) and risk of infection.

The later introduction of the mRNA-1273 vaccine for the UK booster campaign meant that the matched cohort contained younger and healthier people than the whole population receiving a third dose, owing to the prioritisation of older people and those at higher risk. For example, the number of people with immunosuppressive conditions in the matched cohort was just 1.5%. The applicability of our findings to the most vulnerable groups may therefore be limited. However, numbers remained sufficient to compare subgroups defined by age, clinical vulnerability, and previous infection status, including for covid-19 related hospital admission.

To control for spatiotemporal heterogeneity in risk of infection during the study period, we matched on the date of receipt of third dose and local health administration (STP). Some residual heterogeneity within STP regions is possible, but more precise geographical matching was not feasible owing to poor matching success at this level, partly due to time periods during which only one vaccine type was available within a small geographical area.

Some outcomes may be under-ascertained. In particular, positive SARS-CoV-2 tests include only those reported via the national covid-19 surveillance system (SGSS), so many asymptomatic and some symptomatic infections will have been missed. Post-baseline SARS-CoV-2 testing rates were slightly higher in mRNA-1273 than in BNT162b2 recipients, which largely rules out differences in testing behaviours as an explanation for mRNA-1273’s apparent superiority for preventing positive SARS-CoV-2 tests.

This study does not include anyone who received mRNA-1273 as a primary course owing to low numbers of recipients of this vaccine as a primary course. We were therefore unable to assess whether the relative benefit of mRNA-1273 compared with BNT162b2 arose because of heterologous boosting with a different vaccine brand, as BNT162b2 booster recipients had received either BNT162b2 or ChAdOx1first and second vaccine doses. BNT162b2 might outperform mRNA-1273 boosting in people who had received mRNA-1273 first and second doses. However, subgroup analyses in those who received ChAdOx1 first and second doses also found relative benefit of mRNA-1273 over BNT162b2 boosting.

### Findings in context

The COV-BOOST phase 2 randomised trial assessed the safety and immunogenicity of seven covid-19 vaccines for boosting, including BNT162b2 and mRNA-1273, against a menACWY control in people with no previous history of infection.^7^ It found higher immunogenicity of mRNA-1273 compared with BNT162b2 in subgroups with a two dose BNT162b2 primary course (geometric mean ratio of SARS-CoV-2 anti-spike IgG versus control 11.49 (95% confidence interval 9.36 to 14.12) versus (6.78 (5.51 to 8.35)) and a two dose ChAdOx1 primary course (32.30 (24.84 to 42.01) versus 16.80 (12.97 to 21.76)). Likewise, the MixNMatch phase 1-2 non-randomised trial documented higher antibody concentrations after mRNA-1273 boost than BNT162b2 boost among people who received a primary course of BNT162b2.^8^ These trials did not evaluate efficacy endpoints, and we are aware of no other planned or published randomised trials making direct comparisons of boosting with BNT162b2 versus mRNA-1273.

A study in Spanish registry data of people aged 40 years or over with no previous positive test for SARS-CoV-2 examined effectiveness of mRNA vaccine boosting, including a comparison of BNT162b2 with mRNA-1273.^9^ For positive SARS-CoV-2 tests, it estimated a 34 day risk difference comparing mRNA-1273 with BNT162b2 of −2.2 (−2.7 to−1.6) per 1000 people (risk ratio 0.87, 0.84, 0.90). Effectiveness against hospital admission or other severe outcomes was not assessed. A study in the US Veteran Affairs healthcare system in the alpha and delta variant eras using a similar matching approach,^10^ with 65 196 matched recipients in each vaccine group, reported a 16 week risk ratio of 0.87 (0.77 to 0.94) for documented SARS-CoV-2 infection, 0.61 (0.36 to 0.79) for covid-19 related hospital admission, and 0.92 (0.16 to 2.17) for covid-19 related death.

Comparisons of vaccines for primary vaccination in observational data have also been made. Another US Veteran Affairs study by the same group during the alpha period identified an additional 1.23 (95% confidence interval 0.72 to 1.81) documented infections per 1000 people in those with a BNT162b2 first dose compared with an mRNA-1273 first dose at 24 weeks, 0.55 (0.36 to 0.83) covid-19 related hospital admission, 0.10 (0.00 to 0.26) covid-19 related intensive care unit admissions, and 0.02 (−0.06 to 0.12) covid-19 related deaths.^11^ Another study using Veterans Affairs data with exact matching and propensity matching in a similar time period estimated similar differences at 24 weeks for documented infection (1.73 (1.50 to 1.96) additional events per 1000 people in those with a BNT162b2 first dose compared with mRNA-1273), covid-19 related hospital admission (0.56 (0.45 to 0.67), and covid-19 related death (0.03, −0.00 to 0.07).^12^ Accounting for the longer follow-up period and statistical uncertainty, these estimates are consistent with our findings for the comparative effectiveness of mRNA-1273 versus BNT162b2 boosting.

A study using English data to estimate the effectiveness of BNT162b2 boosting versus no boosting and mRNA-1273 boosting versus no boosting separately using a test negative-control design suggests marginally higher effectiveness against symptomatic disease for mRNA-1273 than BNT162b2, but this assumes that the respective control populations in each analysis were similar.^13^

The evidence therefore strongly points to a benefit of mRNA-1273 over BNT162b2 for primary vaccination and subsequent booster doses. This is relevant to vaccine procurement decisions for future booster programmes. However, both vaccines are safe and strongly effective against infection and covid-19, compared with no boosting.^9^ ^13^ ^14^ ^15^ ^16^ ^17^ ^18^ ^19^ Findings from this study should not discourage people from receiving BNT162b2 booster vaccination if offered.

### Conclusions

Covid-19-related outcomes after vaccination with BNT162b2 or mRNA-1273 as a third dose were rare, although risks were estimated to be higher for BNT162b2 than for mRNA-1273. These findings were broadly consistent across subgroups. These constitute important differences at a population level, but either vaccine is preferable compared with no booster dose.

## What is already known on this topic

Both BNT162b2 and mRNA-1273 vaccines are known to provide some protection against infection and severe covid-19 related outcomes when used for boosting
No trials have directly compared the effectiveness of these vaccines with sufficient power to study rare, patient centred outcomes such as hospital admission or death

## What this study adds

This study exploits the concurrent roll-out of both vaccines in the UK booster campaign to compare vaccines for boosting in more than 3 million people
It estimated a marginal benefit of mRNA-1273 compared with BNT162b2 against positive SARS-CoV-2 tests and covid-19 related hospital admission at 20 weeks after receipt of the booster dose

## Data Availability

Access to the underlying identifiable and potentially re-identifiable pseudonymised electronic health record data are tightly governed by various legislative and regulatory frameworks and restricted by best practice. The data in OpenSAFELY are drawn from general practice data across England where TPP is the data processor. TPP developers initiate an automated process to create pseudonymised records in the core OpenSAFELY database, which are copies of key structured data tables in the identifiable records. These pseudonymised records are linked onto key external data resources that have also been pseudonymised via SHA-512 one-way hashing of NHS numbers using a shared salt. Bennett Institute for Applied Data Science developers and principal investigators holding contracts with NHS England have access to the OpenSAFELY pseudonymised data tables as needed to develop the OpenSAFELY tools. These tools in turn enable researchers with OpenSAFELY data access agreements to write and execute code for data management and data analysis without direct access to the underlying raw pseudonymised patient data and to review the outputs of this code. All code for the full data management pipeline—from raw data to completed results for this analysis—and for the OpenSAFELY platform as a whole is available for review at github.com/OpenSAFELY.

## Web extra

Extra material supplied by authors

Web appendix: Supplementary materials

## Notes

Contributors: WJH, EMFH, EPKP, RHK, EJW, VW, TP, BG, MAH, and JACS conceptualised the study. WJH, HJC, AW, AM, BM, CDA, and SCJB were responsible for data curation. WJH, EMFH, EPKP, and VW did the formal analysis. JM, BG, and JACS acquired funding. WJH, EMFH, EPKP, RHK, EJW, VW, TP, MAH, and JACS were responsible for the methodology. WJH, AM, JM, BG, CDA, and JACS were responsible for project administration. WJH, HJC, AW, AM, JM, BM, SCJB, and BG were responsible for resources. WJH, EMFH, EPKP, and SCJB were responsible for software. MAH and JACS provided supervision. WJH, EMFH, EPKP, CDA, and VW were responsible for validation. WJH, EMFH, and EPKP were responsible for visualisation. WJH wrote the original draft of the manuscript. WJH, EMFH, EPKP, RHK, EJW, VW, TP, HJC, AW, CDA, AM, JM, BM, SCJB, BG, MAH, and JACS reviewed and edited the manuscript. MAH and JAAC are joint senior authors. JACS is the guarantor. The corresponding author attests that all listed authors meet authorship criteria and that no others meeting the criteria have been omitted

Information governance: NHS England is the data controller for OpenSAFELY-TPP, TPP is the data processor, and study authors using OpenSAFELY have the approval of NHS England. This implementation of OpenSAFELY is hosted within the TPP environment, which is accredited to the ISO 27001 information security standard and is NHS IG Toolkit compliant.^20^ Patients’ data have been pseudonymised for analysis and linkage using industry standard cryptographic hashing techniques; all pseudonymised datasets transmitted for linkage onto OpenSAFELY are encrypted; access to the platform is via a virtual private network (VPN) connection, restricted to a small group of researchers; the researchers hold contracts with NHS England and access the platform only to initiate database queries and statistical models; all database activity is logged; only aggregate statistical outputs leave the platform environment following best practice for anonymisation of results such as statistical disclosure control for low cell counts.^21^ The OpenSAFELY research platform adheres to the obligations of the UK General Data Protection Regulation and the Data Protection Act 2018. In March 2020 the Secretary of State for Health and Social Care used powers under the UK Health Service (Control of Patient Information) Regulations 2002 to require organisations to process confidential patient information for the purposes of protecting public health, providing healthcare services to the public and monitoring and managing the covid-19 outbreak and incidents of exposure; this sets aside the requirement for patients’ consent.^22^ Taken together, these provide the legal bases to link patient datasets on the OpenSAFELY platform. General practices, from which the primary care data are obtained, are required to share relevant health information to support the public health response to the pandemic, and have been informed of the OpenSAFELY analytics platform.

The lead author (the manuscript’s guarantor) affirms that this manuscript is an honest, accurate, and transparent account of the study being reported; that no important aspects of the study have been omitted; and that any discrepancies from the study as planned (and, if relevant, registered) have been explained.

Dissemination to participants and related patient and public communities: We have developed a website (https://opensafely.org/) through which we invite any member of the public to contact us about this study or the broader OpenSAFELY project.

Provenance and peer review: Not commissioned; externally peer reviewed.

## References

[1] NHS England. NHS begins COVID-19 booster vaccination campaign. 2021. https://www.england.nhs.uk/2021/09/nhs-begins-covid-19-booster-vaccination-campaign/.

[2] UK Health Security Agency. COVID-19: the green book, chapter 14a. 2022. https://www.gov.uk/government/publications/covid-19-the-green-book-chapter-14a.

[3] NHS England. NHS booster bookings open to every eligible adult. 2021. https://www.england.nhs.uk/2021/12/nhs-booster-bookings-open-to-every-eligible-adult/.

[4] NHS England. NHS people 40 and over to get their lifesaving booster jab three months on from second dose. 2021. https://www.england.nhs.uk/2021/12/people-40-and-over-to-get-their-lifesaving-booster-jab-three-months-on-from-second-dose/.

[5] NHS England. NHS to roll out life-saving booster jab to people aged 30-plus. 2021. https://www.england.nhs.uk/2021/12/nhs-to-roll-out-life-saving-booster-jab-to-people-aged-30-plus/.

[6] Lipsitch M Tchetgen Tchetgen E Cohen T . Negative controls: a tool for detecting confounding and bias in observational studies. Epidemiology 2010;21:383-8. 10.1097/EDE.0b013e3181d61eeb 20335814 PMC3053408

[7] Munro APS Janani L Cornelius V COV-BOOST study group . Safety and immunogenicity of seven COVID-19 vaccines as a third dose (booster) following two doses of ChAdOx1 nCov-19 or BNT162b2 in the UK (COV-BOOST): a blinded, multicentre, randomised, controlled, phase 2 trial. Lancet 2021;398:2258-76. 10.1016/S0140-6736(21)02717-3 34863358 PMC8639161

[8] Atmar RL Lyke KE Deming ME DMID 21-0012 Study Group . Homologous and Heterologous Covid-19 Booster Vaccinations. N Engl J Med 2022;386:1046-57. 10.1056/NEJMoa2116414 35081293 PMC8820244

[9] Monge S Rojas-Benedicto A Olmedo C IBERCovid . Effectiveness of mRNA vaccine boosters against infection with the SARS-CoV-2 omicron (B.1.1.529) variant in Spain: a nationwide cohort study. Lancet Infect Dis 2022;22:1313-20. 10.1016/S1473-3099(22)00292-4 35658998 PMC9162477

[10] Dickerman BA Gerlovin H Madenci AL . Comparative effectiveness of third doses of mRNA-based COVID-19 vaccines in US veterans. Nat Microbiol 2023;8:55-63. 10.1038/s41564-022-01272-z 36593297 PMC9949349

[11] Dickerman BA Gerlovin H Madenci AL . Comparative Effectiveness of BNT162b2 and mRNA-1273 Vaccines in U.S. Veterans. N Engl J Med 2022;386:105-15. 10.1056/NEJMoa2115463 34942066 PMC8693691

[12] Ioannou GN Locke ER Green PK Berry K . Comparison of Moderna versus Pfizer-BioNTech COVID-19 vaccine outcomes: A target trial emulation study in the U.S. Veterans Affairs healthcare system. EClinicalMedicine 2022;45:101326. 10.1016/j.eclinm.2022.101326 35261970 PMC8896984

[13] Andrews N Stowe J Kirsebom F . Effectiveness of COVID-19 booster vaccines against COVID-19-related symptoms, hospitalization and death in England. Nat Med 2022;28:831-7. 10.1038/s41591-022-01699-1 35045566 PMC9018410

[14] Barda N Dagan N Ben-Shlomo Y . Safety of the BNT162b2 mRNA Covid-19 Vaccine in a Nationwide Setting. N Engl J Med 2021;385:1078-90. 10.1056/NEJMoa2110475 34432976 PMC8427535

[15] Klein NP Lewis N Goddard K . Surveillance for Adverse Events After COVID-19 mRNA Vaccination. JAMA 2021;326:1390-9. 10.1001/jama.2021.15072 34477808 PMC8511971

[16] Diaz GA Parsons GT Gering SK Meier AR Hutchinson IV Robicsek A . Myocarditis and Pericarditis After Vaccination for COVID-19. JAMA 2021;326:1210-2. 10.1001/jama.2021.13443 34347001 PMC8340007

[17] Dickerman BA Madenci AL Gerlovin H . Comparative Safety of BNT162b2 and mRNA-1273 Vaccines in a Nationwide Cohort of US Veterans. JAMA Intern Med 2022;182:739-46. 10.1001/jamainternmed.2022.2109 35696161 PMC9194743

[18] Hulme WJ Williamson EJ Horne E . Effectiveness of BNT162b2 booster doses in England: An observational study in OpenSAFELY-TPP. medRxiv 2022. 10.1101/2022.06.06.22276026. PMC11191555 38912714

[19] Barda N Dagan N Cohen C . Effectiveness of a third dose of the BNT162b2 mRNA COVID-19 vaccine for preventing severe outcomes in Israel: an observational study. Lancet 2021;398:2093-100. 10.1016/S0140-6736(21)02249-2 34756184 PMC8555967

[20] NHS Digital. Data Security and Protection Toolkit. https://digital.nhs.uk/data-and-information/looking-after-information/data-security-and-information-governance/data-security-and-protection-toolkit.

[21] NHS Digital. ISB1523: Anonymisation Standard for Publishing Health and Social Care Data. https://digital.nhs.uk/data-and-information/information-standards/information-standards-and-data-collections-including-extractions/publications-and-notifications/standards-and-collections/isb1523-anonymisation-standard-for-publishing-health-and-social-care-data.

[22] Department of Health and Social Care. Coronavirus (COVID-19): Notification to organisations to share information. 2020. https://web.archive.org/web/20200421171727/https://www.gov.uk/government/publications/coronavirus-covid-19-notification-of-data-controllers-to-share-information.

