# Supplementary material for: Comparative effectiveness of BNT162b2 versus mRNA-1273 covid-19 vaccine boosting in England: matched cohort study in OpenSAFELY-TPP
Source: BMJ. 2023 Mar 15;380:e072808. doi: 10.1136/bmj-2022-072808 (PMC10014664; doi:10.1136/bmj-2022-072808)
Supplement: Supplementary file 1 — Web appendix: Supplementary materials [file hulw072808.ww1.pdf]

Supplementary materials for Comparative effectiveness of BNT162b2 versus mRNA-1273 boosting in England

Figure S1: Cumulative numbers included in the matched sample

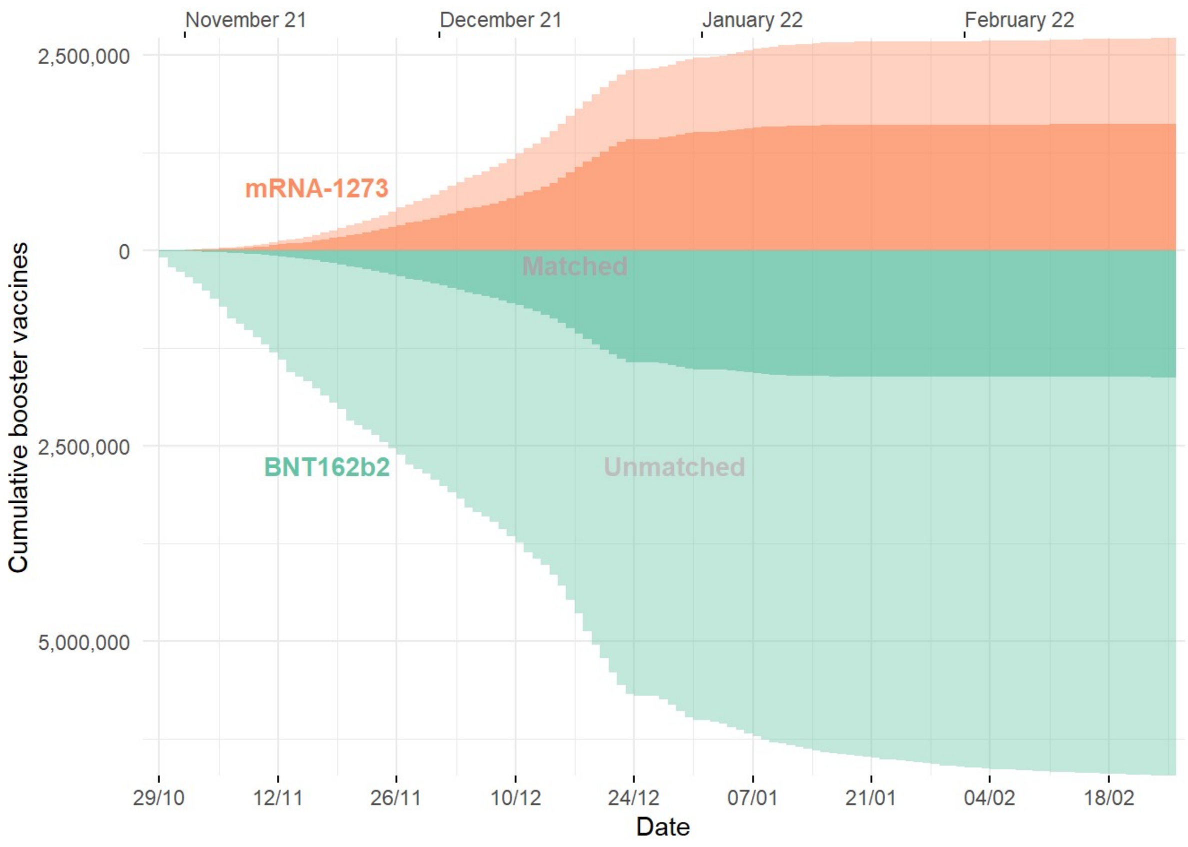

Table S1: Baseline characteristics between BNT12b2 and mRNA-1273 groups, before and after matching

		Before matching			After matching		
**Variable**		**BNT162b2 (N=6721857)**	**mRNA-1273 (N=2719653)**	**SMD**	**BNT162b2 (N=1618959)**	**mRNA-1273 (N=1618959)**	**SMD**
NA		NA	NA	NA	NA	NA	NA
NA	BNT162b2-BNT162b2	2553483 (38.0)	816675 (30.0)	-0.169	476211 (29.4)	476211 (29.4)	0.000
	ChAdOx1-ChAdOx1	4168377 (62.0)	1902981 (70.0)	0.169	1142745 (70.6)	1142745 (70.6)	0.000
Days between dose 2 and 3		188.9 (31.0)	184.9 (29.9)	-0.130	179.3 (26.9)	179.2 (26.8)	-0.005
Age		53.1 (16.4)	49.6 (14.2)	-0.225	49.3 (13.8)	49.3 (13.8)	0.000

Variable		Before matching			After matching		
		BNT162b2 (N=6721857)	mRNA-1273 (N=2719653)	SMD	BNT162b2 (N=1618959)	mRNA-1273 (N=1618959)	SMD
Age band	18-39	1711407 (25.5)	752691 (27.7)	0.050	448437 (27.7)	448437 (27.7)	0.000
	40-49	1126899 (16.8)	586551 (21.6)	0.122	344865 (21.3)	344865 (21.3)	0.000
	50-54	728643 (10.8)	380973 (14.0)	0.096	238179 (14.7)	238179 (14.7)	0.000
	55-59	750813 (11.2)	379107 (13.9)	0.084	235779 (14.6)	235779 (14.6)	0.000
	60-64	703851 (10.5)	300045 (11.0)	0.018	183423 (11.3)	183423 (11.3)	0.000
	65-69	685179 (10.2)	173217 (6.4)	-0.139	102165 (6.3)	102165 (6.3)	0.000
	70-74	575607 (8.6)	89001 (3.3)	-0.226	49305 (3.0)	49305 (3.0)	0.000
	75-79	263205 (3.9)	34965 (1.3)	-0.166	13011 (0.8)	13011 (0.8)	0.000
	80+	176247 (2.6)	23091 (0.8)	-0.136	3789 (0.2)	3789 (0.2)	0.000
Sex	Female	3425607 (51.0)	1321665 (48.6)	-0.047	770097 (47.6)	770097 (47.6)	0.000
	Male	3296253 (49.0)	1397991 (51.4)	0.047	848859 (52.4)	848859 (52.4)	0.000
Ethnicity	White	5704521 (84.9)	2310945 (85.0)	0.003	1379673 (85.2)	1378173 (85.1)	-0.003
	Black	85809 (1.3)	31071 (1.1)	-0.012	14901 (0.9)	14565 (0.9)	-0.002
	South Asian	372783 (5.5)	126777 (4.7)	-0.040	70923 (4.4)	67365 (4.2)	-0.011
	Mixed	57723 (0.9)	23577 (0.9)	0.001	12993 (0.8)	13119 (0.8)	0.001
	Other	110115 (1.6)	44133 (1.6)	-0.001	24375 (1.5)	24939 (1.5)	0.003
	Unknown	390903 (5.8)	183147 (6.7)	0.038	116085 (7.2)	120795 (7.5)	0.011
Deprivation	1 (most deprived)	1041159 (15.5)	376563 (13.8)	-0.046	196185 (12.1)	196185 (12.1)	0.000
	2	1249545 (18.6)	482193 (17.7)	-0.022	277887 (17.2)	277887 (17.2)	0.000
	3	1512507 (22.5)	601221 (22.1)	-0.009	371733 (23.0)	371733 (23.0)	0.000
	4	1497663 (22.3)	621525 (22.9)	0.014	388203 (24.0)	388203 (24.0)	0.000
	5 (least deprived)	1420989 (21.1)	638145 (23.5)	0.056	384951 (23.8)	384951 (23.8)	0.000
Region	North East and Yorkshire	1190103 (17.7)	499473 (18.4)	0.017	327741 (20.2)	327741 (20.2)	0.000
	North West	530145 (7.9)	312927 (11.5)	0.123	162225 (10.0)	162225 (10.0)	0.000
	Midlands	1507203 (22.4)	529257 (19.5)	-0.073	307041 (19.0)	307041 (19.0)	0.000
	East of England	1429497 (21.3)	792303 (29.1)	0.182	449895 (27.8)	449895 (27.8)	0.000
	London	336993 (5.0)	157371 (5.8)	0.034	98619 (6.1)	98619 (6.1)	0.000
	South East	556251 (8.3)	90747 (3.3)	-0.212	59103 (3.7)	59103 (3.7)	0.000
	South West	1171659 (17.4)	337575 (12.4)	-0.141	214335 (13.2)	214335 (13.2)	0.000
JCVI clinical risk group	Not clinically at-risk	4615599 (68.7)	2119377 (77.9)	0.211	1403499 (86.7)	1403499 (86.7)	0.000
	Clinically at-risk	1609497 (23.9)	491631 (18.1)	-0.144	199203 (12.3)	199203 (12.3)	0.000
	Clinically extremely vulnerable	496761 (7.4)	108645 (4.0)	-0.147	16257 (1.0)	16257 (1.0)	0.000
Body Mass Index > 40 kg/m^2		280635 (4.2)	104361 (3.8)	-0.017	20691 (1.3)	20661 (1.3)	0.000

Variable		Before matching BNT162b2 (N=6721857)	Before matching mRNA-1273 (N=2719653)	Before matching SMD	After matching BNT162b2 (N=1618959)	After matching mRNA-1273 (N=1618959)	After matching SMD
Chronic heart disease		717747 (10.7)	184023 (6.8)	-0.139	73101 (4.5)	73977 (4.6)	0.003
Chronic kidney disease		279753 (4.2)	53529 (2.0)	-0.128	20493 (1.3)	20223 (1.2)	-0.001
Diabetes		680997 (10.1)	190071 (7.0)	-0.112	65871 (4.1)	65505 (4.0)	-0.001
Chronic liver disease		175137 (2.6)	59673 (2.2)	-0.027	21441 (1.3)	21447 (1.3)	0.000
Chronic respiratory disease		268113 (4.0)	62691 (2.3)	-0.097	20667 (1.3)	20373 (1.3)	-0.002
Asthma		36207 (0.5)	12519 (0.5)	-0.011	3753 (0.2)	3783 (0.2)	0.000
Chronic neurological disease		344535 (5.1)	99909 (3.7)	-0.071	36243 (2.2)	35841 (2.2)	-0.002
Immunosuppressed		143397 (2.1)	31965 (1.2)	-0.075	7017 (0.4)	6333 (0.4)	-0.007
Immunosuppressed (all)		341211 (5.1)	85659 (3.1)	-0.097	24915 (1.5)	24435 (1.5)	-0.002
Asplenia or poor spleen function		49383 (0.7)	15075 (0.6)	-0.023	2805 (0.2)	2817 (0.2)	0.000
Cancer (excluding haem), within previous 3 years		162375 (2.4)	40629 (1.5)	-0.067	15621 (1.0)	15741 (1.0)	0.001
Haematological cancer, within previous 3 years		18399 (0.3)	2979 (0.1)	-0.038	1029 (0.1)	861 (0.1)	-0.004
Solid organ transplant		7197 (0.1)	1245 (0.0)	-0.022	273 (0.0)	225 (0.0)	-0.002
Immunosuppressive medications, within 6 months		60633 (0.9)	12153 (0.4)	-0.056	2865 (0.2)	2391 (0.1)	-0.007
HIV/AIDS		621 (0.0)	123 (0.0)	-0.006	15 (0.0)	21 (0.0)	0.001
Morbidity count	0	4675107 (69.6)	2116401 (77.8)	0.189	1399287 (86.4)	1399287 (86.4)	0.000
	1	1403889 (20.9)	454713 (16.7)	-0.107	177387 (11.0)	177387 (11.0)	0.000
	2+	642861 (9.6)	148533 (5.5)	-0.156	42285 (2.6)	42285 (2.6)	0.000
Learning disabilities		44943 (0.7)	12939 (0.5)	-0.026	2997 (0.2)	2157 (0.1)	-0.013
Serious mental illness		65949 (1.0)	22449 (0.8)	-0.016	4869 (0.3)	4431 (0.3)	-0.005
Number of SARS-CoV-2 tests	0	3395073 (50.5)	1261551 (46.4)	-0.083	786687 (48.6)	758883 (46.9)	-0.034
	1	1251495 (18.6)	528513 (19.4)	0.021	313875 (19.4)	313647 (19.4)	0.000
	2	632631 (9.4)	278289 (10.2)	0.028	158841 (9.8)	162681 (10.0)	0.008
	3+	1442661 (21.5)	651303 (23.9)	0.059	359547 (22.2)	383745 (23.7)	0.036
Prior documented SARS-CoV-2 infection		882525 (13.1)	387447 (14.2)	0.033	149685 (9.2)	149685 (9.2)	0.000

Figure S2: Standardised Mean Differences between BNT12b2 and mRNA-1273 groups, before and after matching

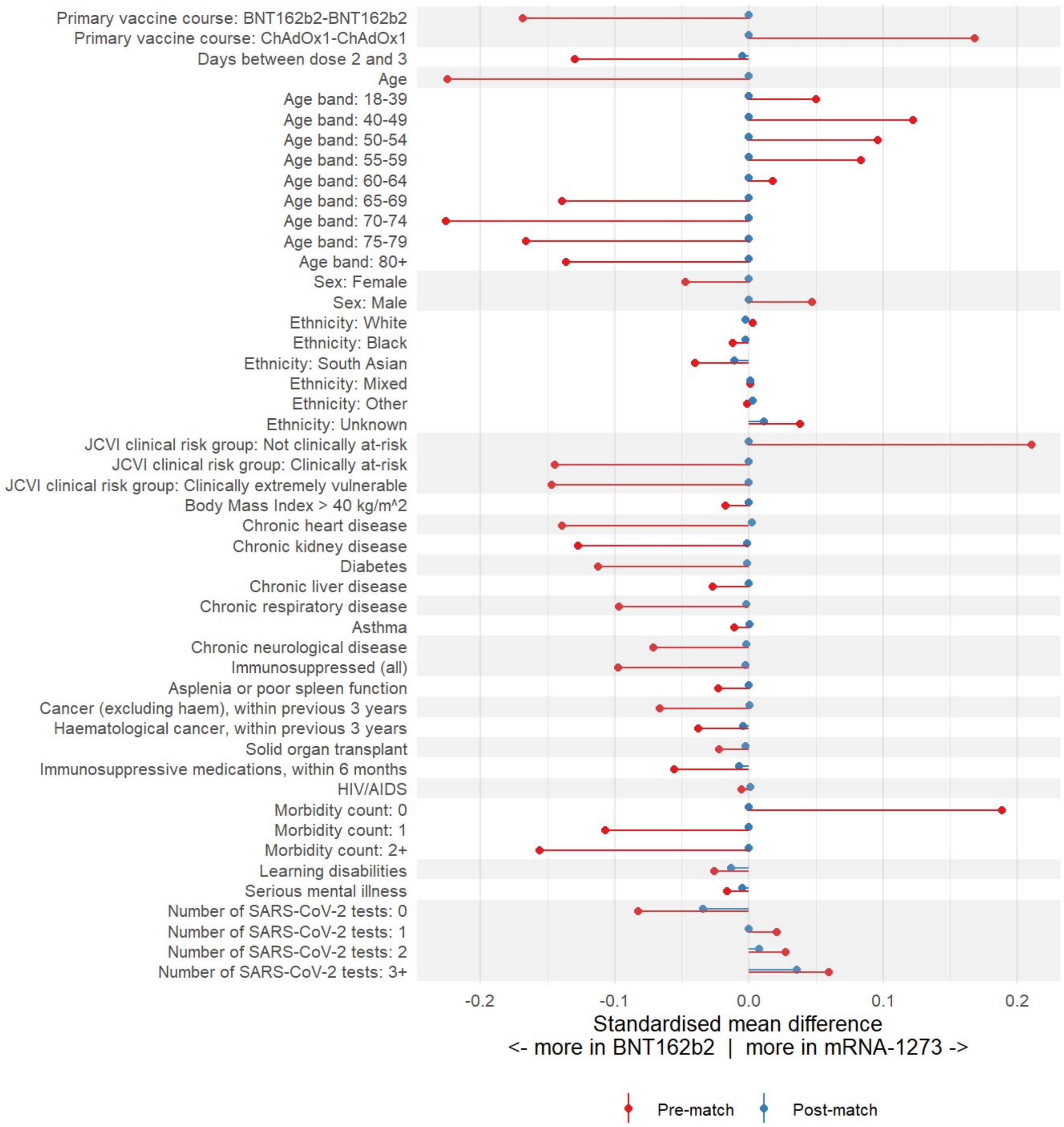

Primary vaccine course: BNT162b2-BNT162b2
Primary vaccine course: ChAdOx1-ChAdOx1
Days between dose 2 and 3
Age
Age band: 18-39
Age band: 40-49
Age band: 50-54
Age band: 55-59
Age band: 60-64
Age band: 65-69
Age band: 70-74
Age band: 75-79
Age band: 80+
Sex: Female
Sex: Male
Ethnicity: White
Ethnicity: Black
Ethnicity: South Asian
Ethnicity: Mixed
Ethnicity: Other
Ethnicity: Unknown
JCVI clinical risk group: Not clinically at-risk
JCVI clinical risk group: Clinically at-risk
JCVI clinical risk group: Clinically extremely vulnerable
Body Mass Index > 40 kg/m^2
Chronic heart disease
Chronic kidney disease
Diabetes
Chronic liver disease
Chronic respiratory disease
Asthma
Chronic neurological disease
Immunosuppressed (all)
Asplenia or poor spleen function
Cancer (excluding haem), within previous 3 years
Haematological cancer, within previous 3 years
Solid organ transplant
Immunosuppressive medications, within 6 months
HIV/AIDS
Morbidity count: 0
Morbidity count: 1
Morbidity count: 2+
Learning disabilities
Serious mental illness
Number of SARS-CoV-2 tests: 0
Number of SARS-CoV-2 tests: 1
Number of SARS-CoV-2 tests: 2
Number of SARS-CoV-2 tests: 3+
-0.2
-0.1
0.0
0.1
0.2
Standardised mean difference
<- more in BNT162b2 | more in mRNA-1273 ->
Pre-match
Post-match

Table S2: 20-week risk differences, risk ratios, and Cox hazard ratios, with p-values for subgroup heterogeneity

Sub-group		Risk difference per 1,000 people (95% CI)	P-value	Risk ratio (95% CI)	P-value	Hazard ratio (95% CI)	P-value
Positive SARS-CoV-2 test							
Main		−4.25 (−5.53 to −2.98)		0.97 (0.97-0.98)		0.95 (0.95 to 0.96)	
Primary vaccine course	ChAdOx1-S	−2.73 (−4.19 to −1.27)	<0.001	0.98 (0.97-0.99)	<0.001	0.96 (0.96 to 0.97)	<0.001
	BNT162b2	−8.73 (−11.3 to −6.12)		0.95 (0.94-0.97)		0.93 (0.92 to 0.94)	
Age-band	18-39	−8.20 (−20.9 to 4.52)	0.578	0.97 (0.92-1.02)	0.724	0.93 (0.92 to 0.94)	<0.001
	40-54	−2.53 (−7.11 to 2.05)		0.99 (0.97-1.01)		0.96 (0.96 to 0.97)	
	55-64	−2.84 (−5.52 to −0.17)		0.98 (0.96-1.00)		0.96 (0.95 to 0.98)	
	65-74	−0.77 (−2.79 to 1.25)		0.99 (0.96-1.02)		0.98 (0.96 to 1.01)	
	75+	−2.71 (−6.42 to 1.00)		0.94 (0.86-1.02)		0.91 (0.83 to 0.99)	
Prior SARS-CoV-2 infection status	No prior SARS-CoV-2 infection	−4.39 (−5.71 to −3.08)	0.873	0.97 (0.97-0.98)	0.365	0.95 (0.95 to 0.96)	0.066
	Prior SARS-CoV-2 infection	−4.82 (−9.86 to 0.23)		0.95 (0.90-1.00)		0.93 (0.90 to 0.96)	
Clinical vulnerability	Not clinically at-risk	−4.48 (−6.07 to −2.90)	0.139	0.97 (0.97-0.98)	0.290	0.95 (0.94 to 0.96)	0.061
	Clinically at-risk	−2.06 (−4.52 to 0.41)		0.98 (0.96-1.00)		0.97 (0.95 to 0.99)	
	Clinically extremely vulnerable	−8.39 (−16.2 to −0.54)		0.93 (0.87-1.00)		0.92 (0.86 to 0.98)	
COVID-19 hospitalisation							
Main		−0.10 (−0.16 to −0.046)		0.86 (0.79-0.94)		0.89 (0.82 to 0.95)	
Primary vaccine course	ChAdOx1-S	−0.100 (−0.17 to −0.030)	0.832	0.87 (0.79-0.96)	0.642	0.89 (0.82 to 0.97)	0.690
	BNT162b2	−0.11 (−0.21 to −0.012)		0.83 (0.71-0.98)		0.86 (0.75 to 1.00)	
Age-band	18-39	−0.055 (−0.14 to 0.028)	0.158	0.86 (0.69-1.08)	0.146	0.86 (0.70 to 1.05)	0.120
	40-54	−0.066 (−0.15 to 0.016)		0.87 (0.74-1.03)		0.88 (0.75 to 1.02)	
	55-64	−0.22 (−0.33 to −0.11)		0.72 (0.62-0.85)		0.77 (0.67 to 0.89)	
	65-74	−0.054 (−0.30 to 0.19)		0.96 (0.81-1.14)		1.01 (0.87 to 1.17)	
	75+	−0.27 (−1.54 to 1.00)		0.95 (0.75-1.20)		0.96 (0.79 to 1.17)	
Prior SARS-CoV-2 infection status	No prior SARS-CoV-2 infection	−0.10 (−0.16 to −0.040)	0.405	0.87 (0.80-0.95)	0.067	0.90 (0.84 to 0.97)	0.047
	Prior SARS-CoV-2 infection	−0.16 (−0.29 to −0.036)		0.58 (0.38-0.89)		0.61 (0.42 to 0.89)	
Clinical vulnerability	Not clinically at-risk	−0.064 (−0.11 to −0.015)	0.060	0.86 (0.77-0.97)	0.837	0.89 (0.81 to 0.98)	0.820
	Clinically at-risk	−0.39 (−0.67 to −0.11)		0.83 (0.72-0.95)		0.86 (0.77 to 0.98)	
	Clinically extremely vulnerable	−0.75 (−2.51 to 1.00)		0.89 (0.68-1.17)		0.94 (0.74 to 1.18)	
COVID-19 death							
Main		−0.004 (−0.015 to 0.007)		0.87 (0.56-1.33)		0.83 (0.58 to 1.19)	
Primary vaccine course	ChAdOx1-S	−0.005 (−0.019 to 0.008)	0.545	0.82 (0.49-1.36)	0.527	0.81 (0.53 to 1.24)	0.822

Sub-group		Risk difference per 1,000 people (95% CI)	P-value	Risk ratio (95% CI)	P-value	Hazard ratio (95% CI)	P-value
	BNT162b2	−0.013 (−0.033 to 0.008)		0.60 (0.26-1.37)		0.89 (0.45 to 1.75)	
Age-band	18-39	0.007 (−0.001 to 0.016)	0.116	Inf (NA-NA)		3.02 (0.31 to 29.06)	0.567
	40-54	−0.011 (−0.023 to 0.001)		0.33 (0.09-1.23)		0.75 (0.26 to 2.16)	
	55-64	0.000 (−0.019 to 0.019)		1.00 (0.40-2.51)		0.66 (0.30 to 1.48)	
	65-74	0.000 (−0.046 to 0.047)		1.01 (0.40-2.54)		0.64 (0.33 to 1.25)	
	75+	−0.24 (−0.61 to 0.14)		0.60 (0.26-1.36)		1.09 (0.60 to 2.00)	
Prior SARS-CoV-2 infection status	No prior SARS-CoV-2 infection	−0.008 (−0.020 to 0.004)	0.030	0.73 (0.47-1.15)		0.83 (0.58 to 1.19)	0.897
	Prior SARS-CoV-2 infection	0.020 (−0.003 to 0.043)		Inf (NA-NA)		1.00 (0.06 to 15.98)	
Clinical vulnerability	Not clinically at-risk	0.000 (−0.008 to 0.008)	0.184	1.00 (0.49-2.04)	0.448	0.90 (0.48 to 1.70)	0.850
	Clinically at-risk	−0.060 (−0.12 to 0.003)		0.56 (0.30-1.04)		0.74 (0.43 to 1.27)	
	Clinically extremely vulnerable	0.000 (−0.30 to 0.30)		1.00 (0.20-4.95)		0.93 (0.45 to 1.93)	
Non-COVID-19 death							
Main		−0.078 (−0.13 to −0.023)		0.88 (0.81-0.96)		0.92 (0.86 to 0.99)	
Primary vaccine course	ChAdOx1-S	−0.063 (−0.13 to 0.006)	0.374	0.91 (0.83-1.01)	0.158	0.94 (0.86 to 1.02)	0.285
	BNT162b2	−0.11 (−0.20 to −0.026)		0.78 (0.65-0.95)		0.85 (0.73 to 0.99)	
Age-band	18-39	0.000 (−0.035 to 0.035)	0.007	1.01 (0.59-1.71)	0.168	1.11 (0.68 to 1.82)	0.065
	40-54	0.010 (−0.050 to 0.070)		1.04 (0.83-1.31)		1.07 (0.88 to 1.30)	
	55-64	−0.098 (−0.21 to 0.010)		0.87 (0.74-1.02)		0.88 (0.77 to 1.01)	
	65-74	−0.15 (−0.44 to 0.14)		0.93 (0.80-1.07)		0.97 (0.86 to 1.09)	
	75+	−2.37 (−3.85 to −0.90)		0.72 (0.59-0.88)		0.76 (0.64 to 0.90)	
Prior SARS-CoV-2 infection status	No prior SARS-CoV-2 infection	−0.073 (−0.13 to −0.014)	0.431	0.90 (0.82-0.98)	0.063	0.93 (0.87 to 1.00)	0.046
	Prior SARS-CoV-2 infection	−0.12 (−0.22 to −0.019)		0.54 (0.32-0.92)		0.59 (0.38 to 0.92)	
Clinical vulnerability	Not clinically at-risk	−0.004 (−0.045 to 0.037)	<0.001	0.99 (0.86-1.13)	0.022	1.02 (0.91 to 1.13)	0.041
	Clinically at-risk	−0.33 (−0.63 to −0.034)		0.87 (0.76-0.99)		0.88 (0.79 to 0.98)	
	Clinically extremely vulnerable	−3.33 (−5.33 to −1.33)		0.67 (0.53-0.85)		0.78 (0.64 to 0.96)	

Table S3: Summary of follow-up time, including time beyond 20 weeks where available

	Weeks of follow-up				
Vaccine	Total	Mean	Median	Q1	Q3
Positive SARS-CoV-2 test					
BNT162b2	24 008 759.6	14.8	15.3	14.3	17.1
mRNA-1273	24 120 247.3	14.9	15.3	14.3	17.1
Both	48 129 006.9	14.9	15.3	14.3	17.1
COVID-19 hospitalisation					
BNT162b2	44 540 421.0	27.5	28.0	27.7	28.0
mRNA-1273	44 508 452.1	27.5	28.0	27.7	28.0
Both	89 048 873.1	27.5	28.0	27.7	28.0
COVID-19 death					
BNT162b2	44 560 983.0	27.5	28.0	27.7	28.0
mRNA-1273	44 525 580.4	27.5	28.0	27.7	28.0
Both	89 086 563.4	27.5	28.0	27.7	28.0
Non-COVID-19 death					
BNT162b2	44 560 983.0	27.5	28.0	27.7	28.0
mRNA-1273	44 525 580.4	27.5	28.0	27.7	28.0
Both	89 086 563.4	27.5	28.0	27.7	28.0

Table S4: Testing rates after baseline, up administrative censoring on 31 March 2022

		BNT162b2			mRNA-1273		
Sub-group		Person-weeks	All tests	Positive tests	Person-weeks	All tests	Positive tests
Main		25 832 089	0.139	0.012	25 833 080	0.151	0.012
Primary vaccine course	ChAdOx1-S	18 712 821	0.147	0.011	18 713 232	0.159	0.011
	BNT162b2	7 119 268	0.121	0.015	7 119 848	0.129	0.014
Age-band	18-39	5 917 556	0.119	0.017	5 885 055	0.125	0.016
	40-54	8 604 362	0.155	0.014	8 624 133	0.170	0.014
	55-64	7 430 046	0.153	0.010	7 464 086	0.165	0.009
	65-74	3 379 514	0.115	0.006	3 355 995	0.126	0.006
	75+	500 612	0.066	0.004	503 811	0.065	0.003
Prior SARS-CoV-2 infection status	No prior SARS-CoV-2 infection	23 580 833	0.140	0.013	23 581 707	0.151	0.013
	Prior SARS-CoV-2 infection	2 251 257	0.135	0.006	2 251 373	0.147	0.006
Clinical vulnerability	Not clinically at-risk	21 998 618	0.143	0.013	21 998 713	0.154	0.012
	Clinically at-risk	3 535 571	0.123	0.009	3 536 059	0.134	0.009
	Clinically extremely vulnerable	297 901	0.109	0.009	298 309	0.111	0.009

Cumulative incidences by subgroup

Figure S3a: Cumulative incidence estimates per 1,000 people

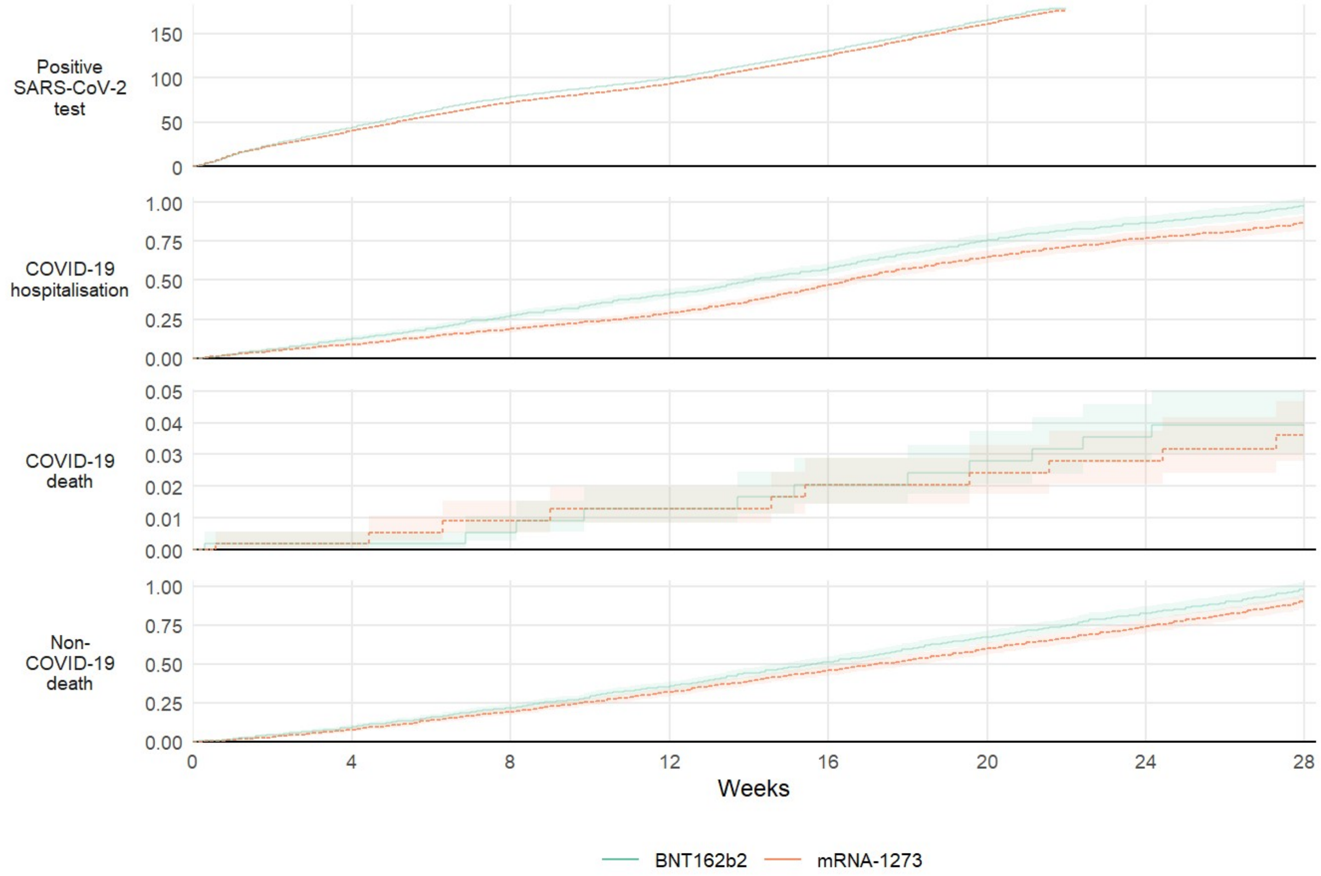

Figure S3b: Cumulative incidence estimates per 1,000 people by primary course type

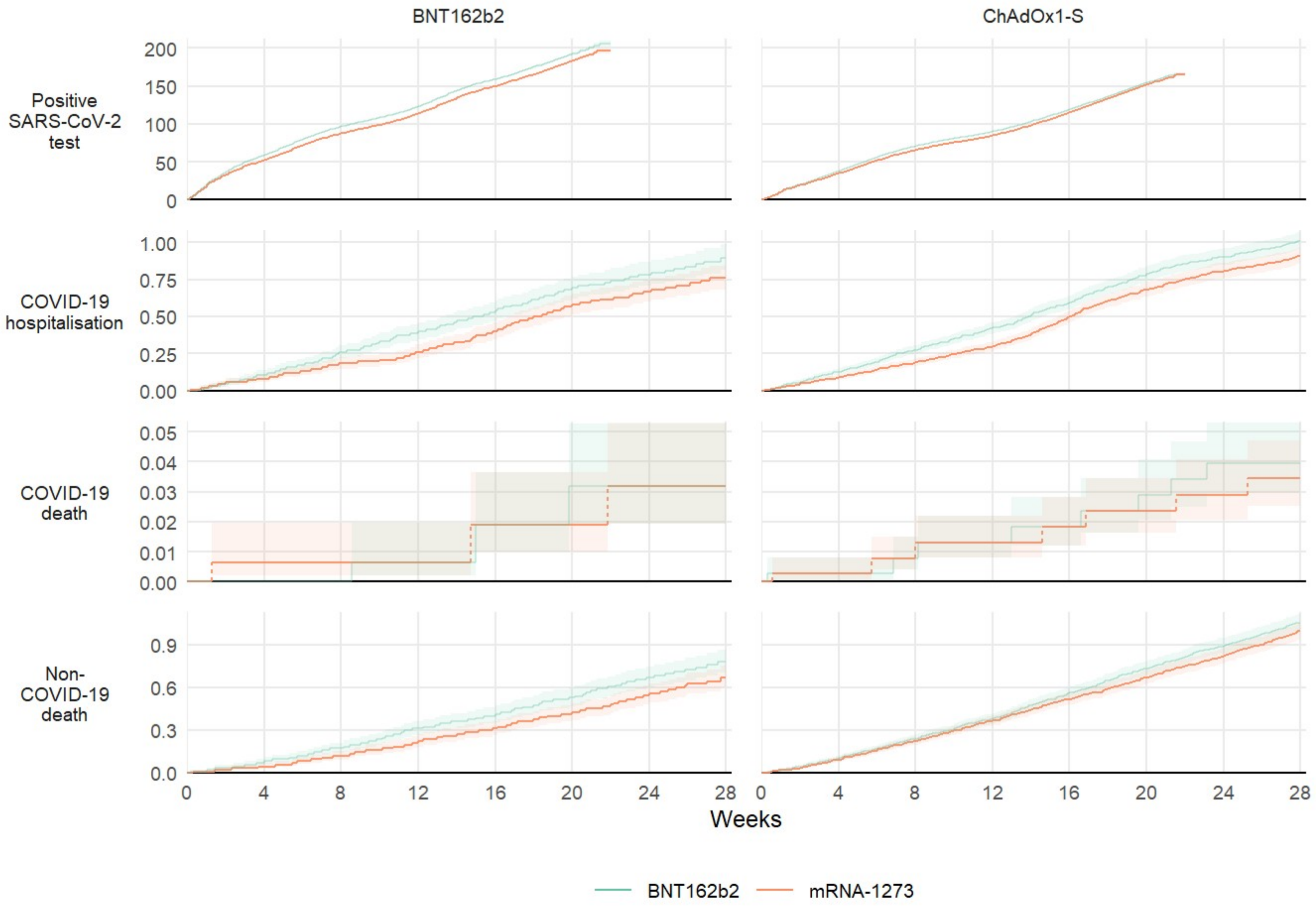

Figure S3c: Cumulative incidence estimates per 1,000 people by age group

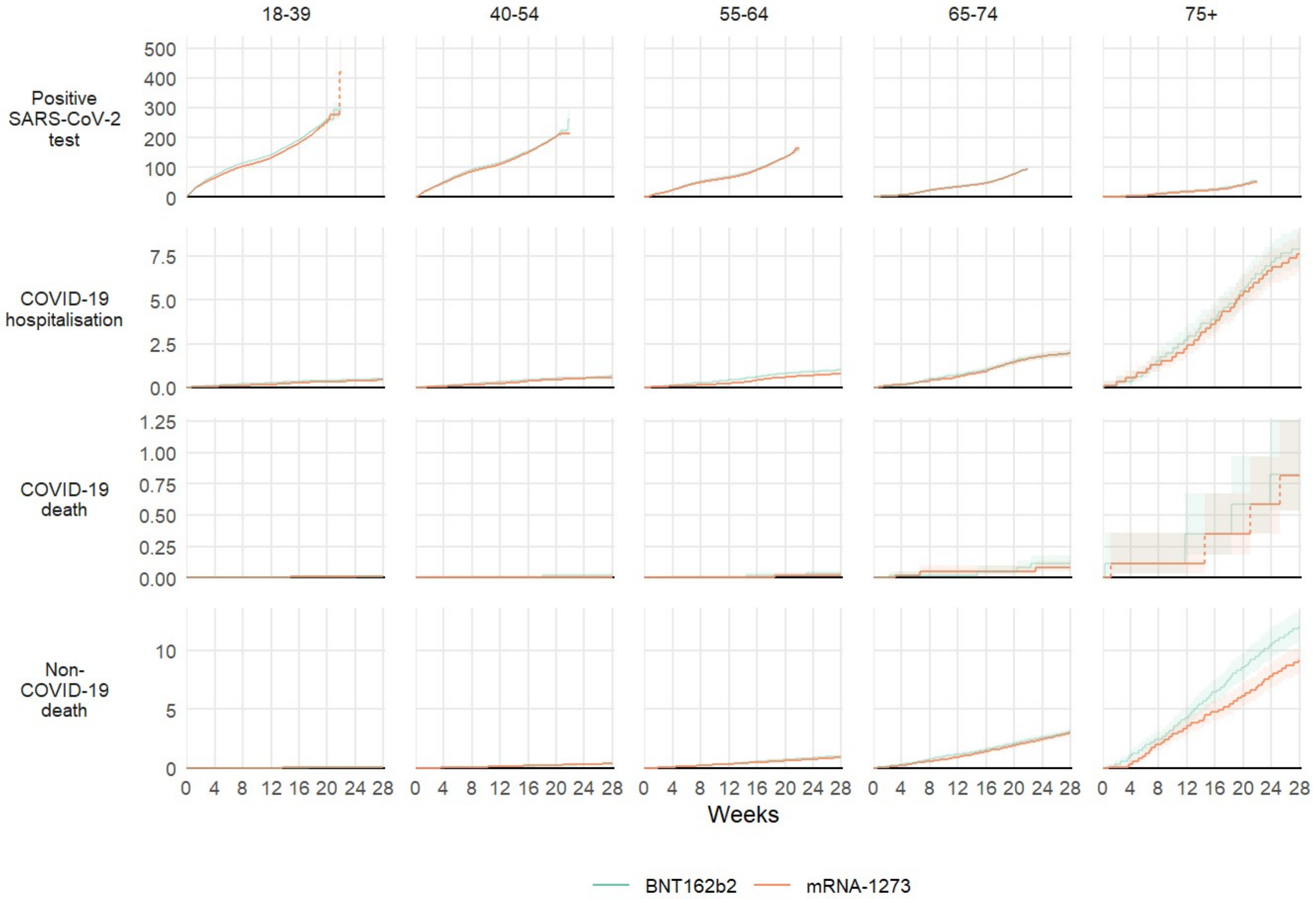

Figure S3d: Cumulative incidence estimates per 1,000 people by prior infection

status

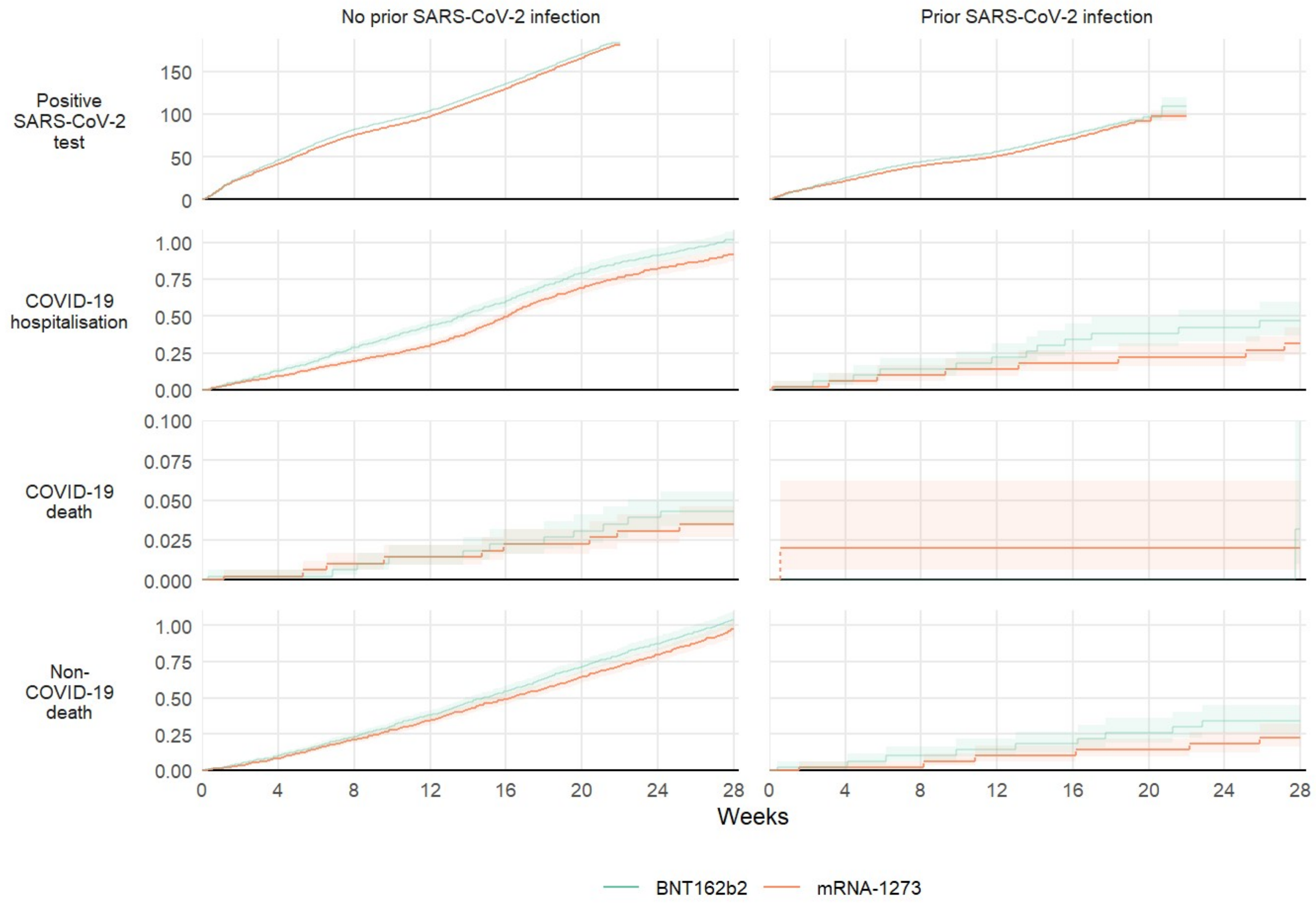

Figure S3e: Cumulative incidence estimates per 1,000 people by clinical vulnerability

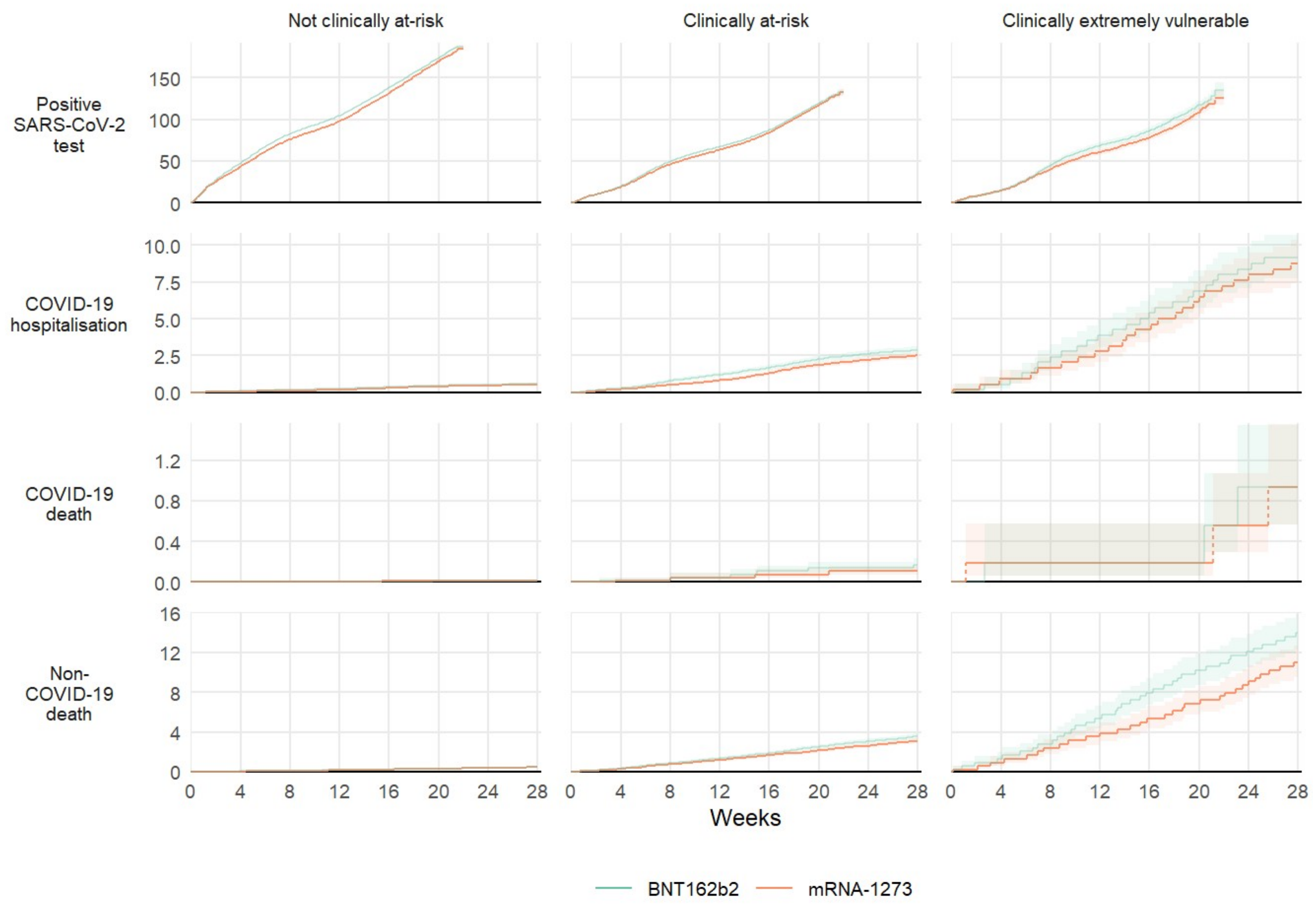

Comparisons by subgroup

Figure S4a: Cumulative risk difference, cumulative risk ratio, and period-specific hazard ratios

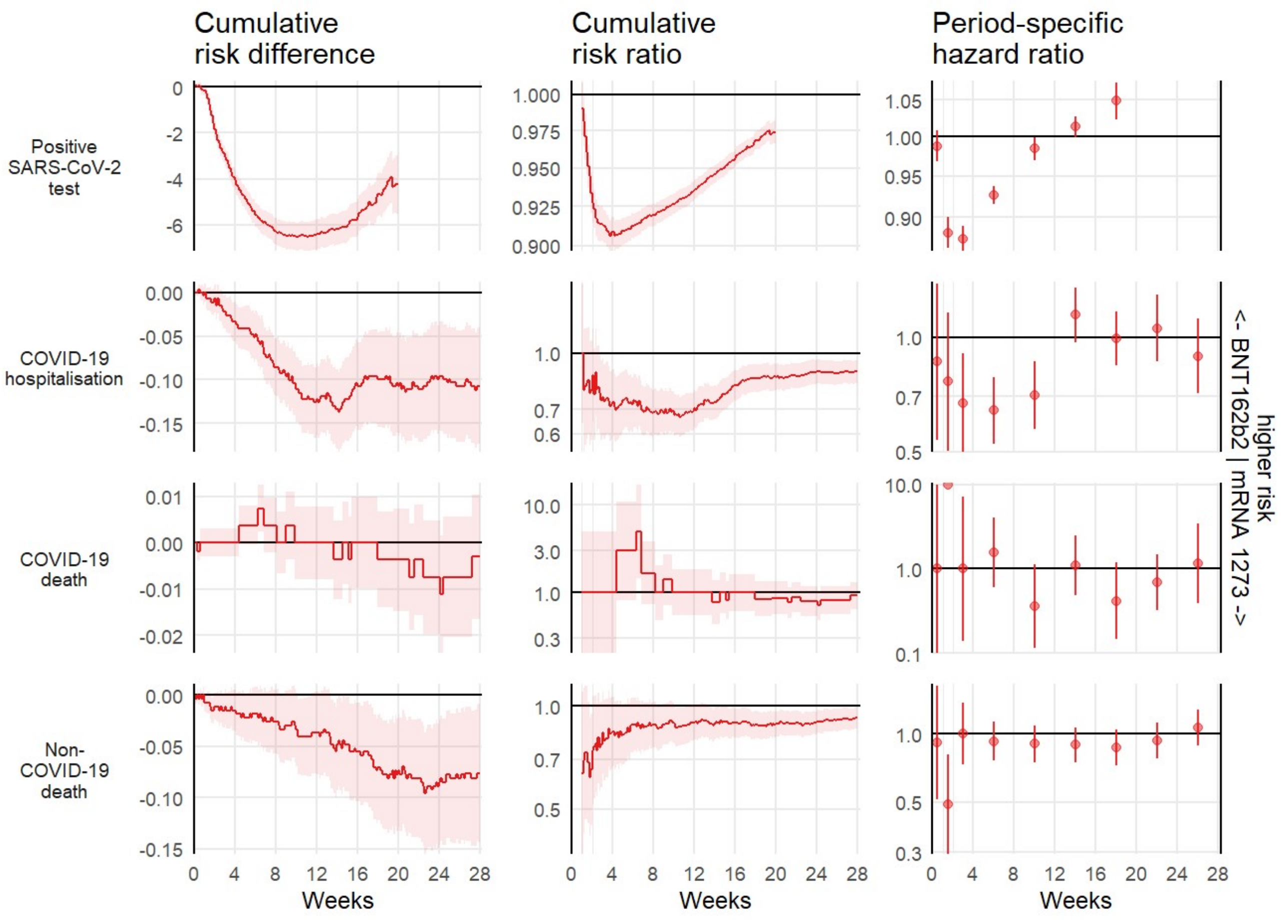

Figure S4b: Cumulative risk difference, cumulative risk ratio, and period-specific hazard ratios by primary course type

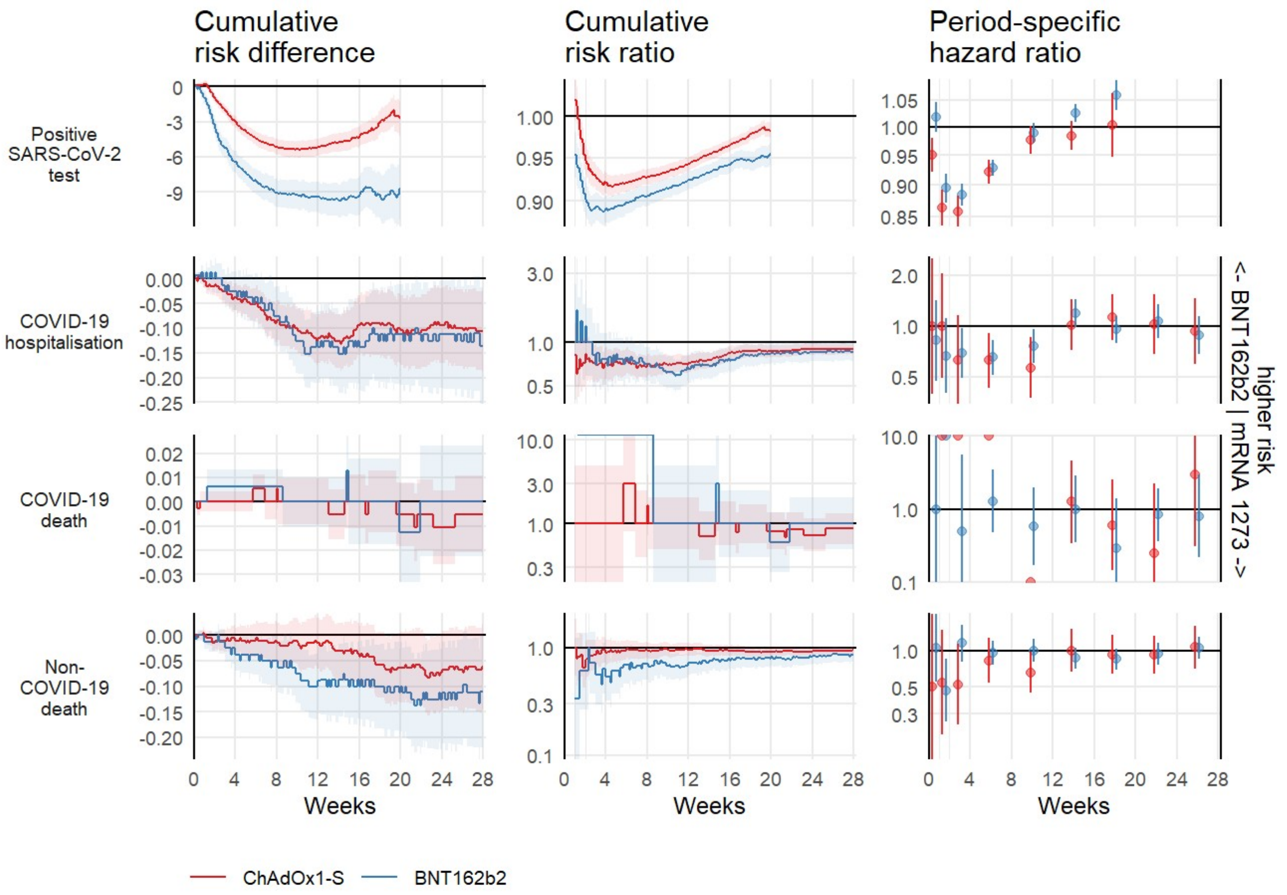

Figure S4c: Cumulative risk difference, cumulative risk ratio, and period-specific hazard ratios by age group

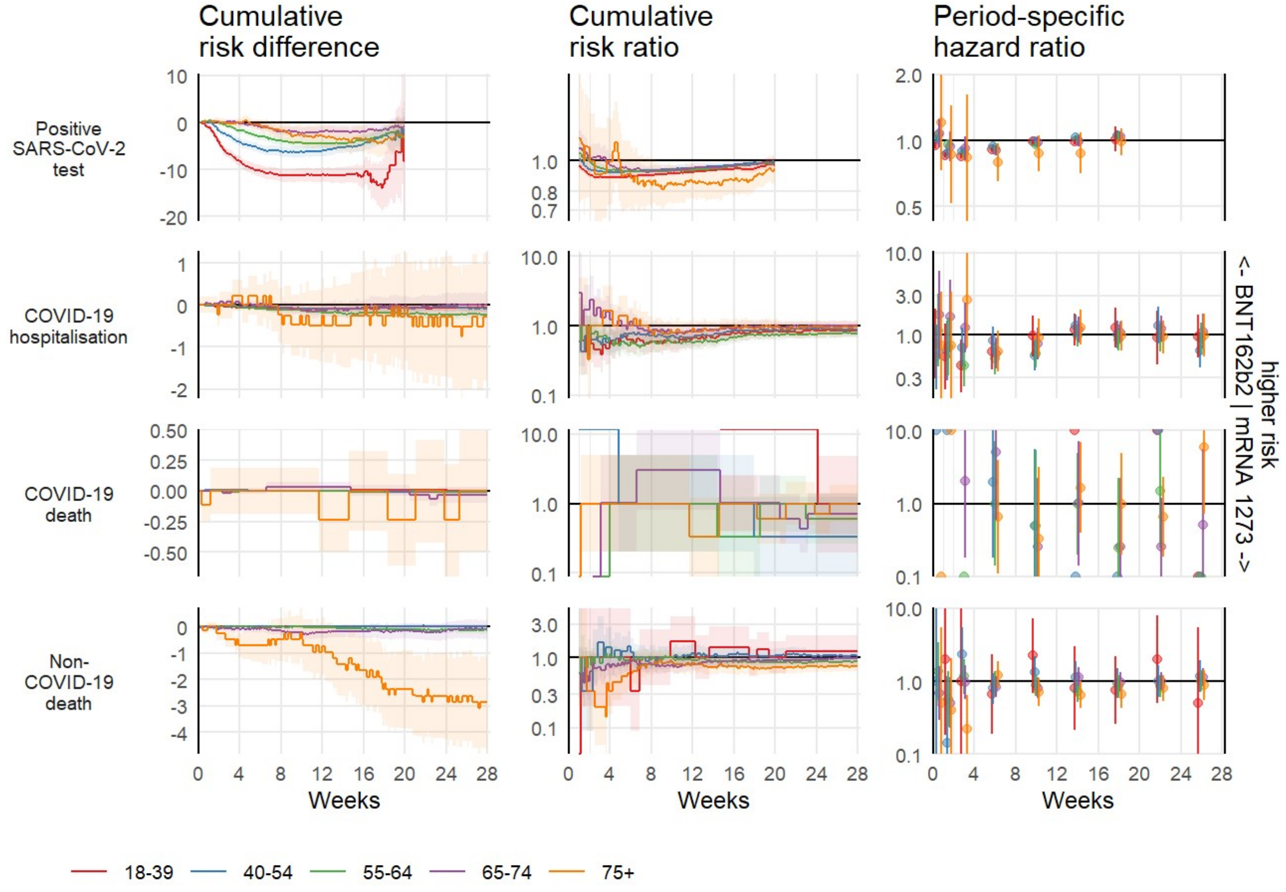

Figure S4d: Cumulative risk difference, cumulative risk ratio, and period-specific hazard ratios by prior infection status

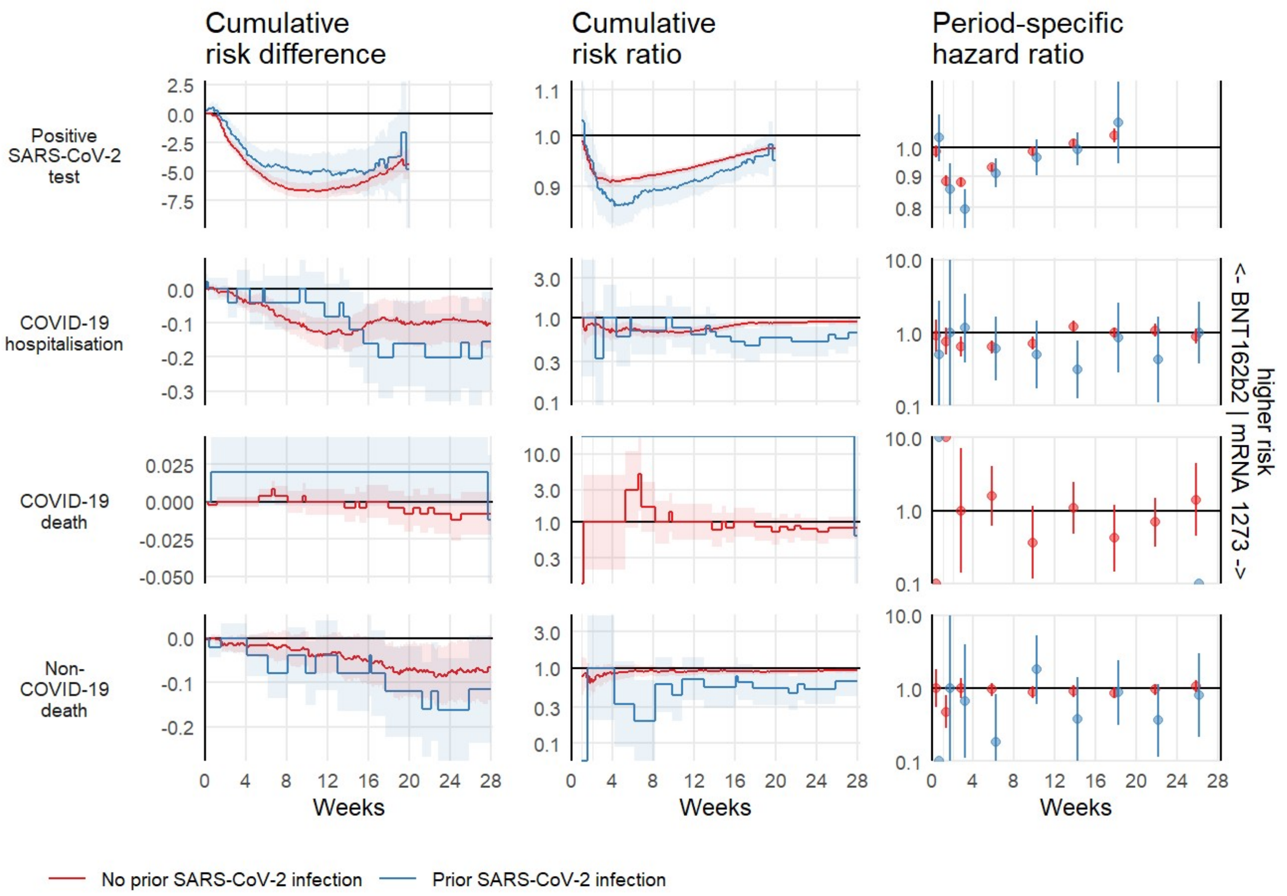

Figure S4e: Cumulative risk difference, cumulative risk ratio, and period-specific hazard ratios by clinical vulnerability

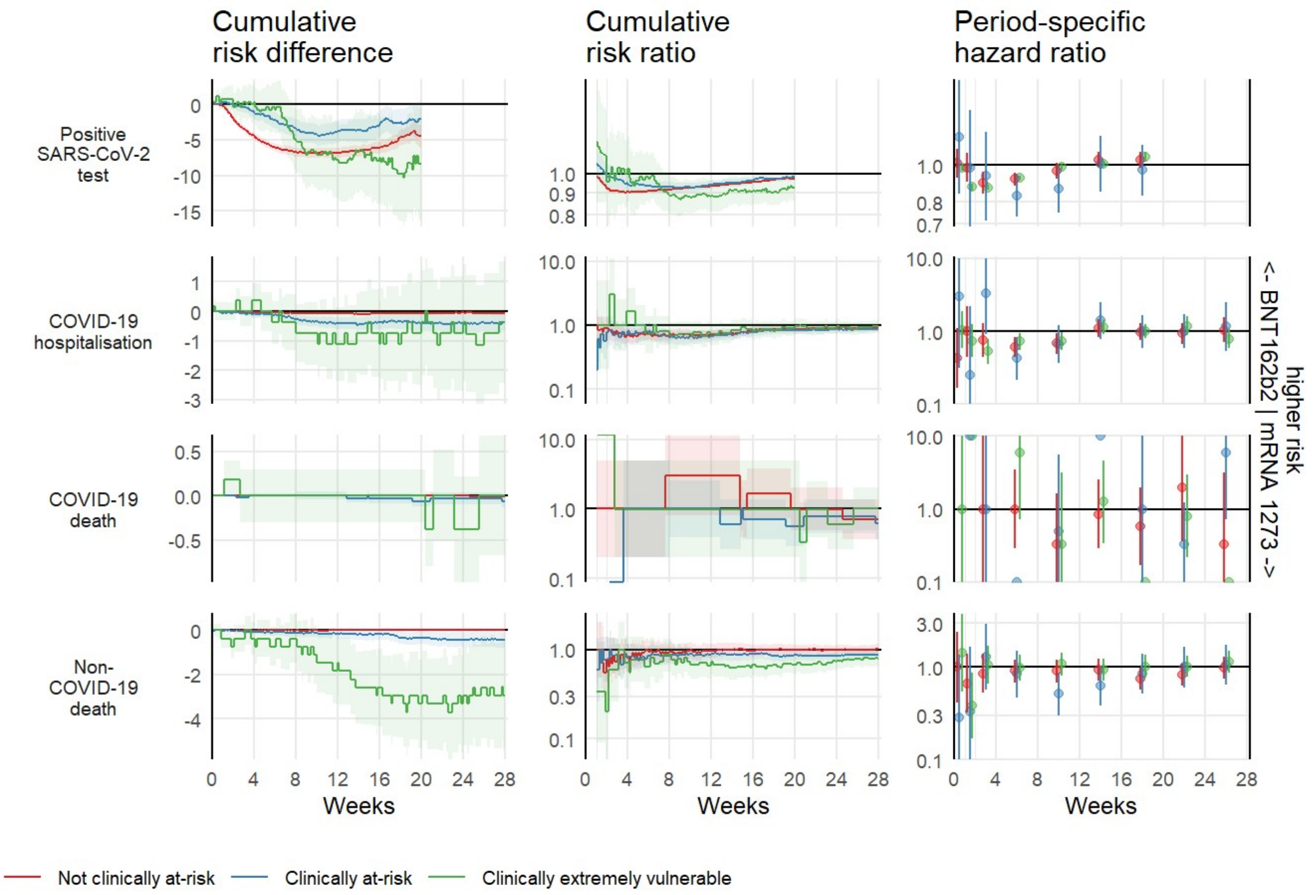

Era-specific comparative effectiveness

Figure S5: 20-week Cox hazard ratios by variant era

Delta is defined as days up to 14 December 2021. Omicron is defined as day from 15 December 2021 onwards.

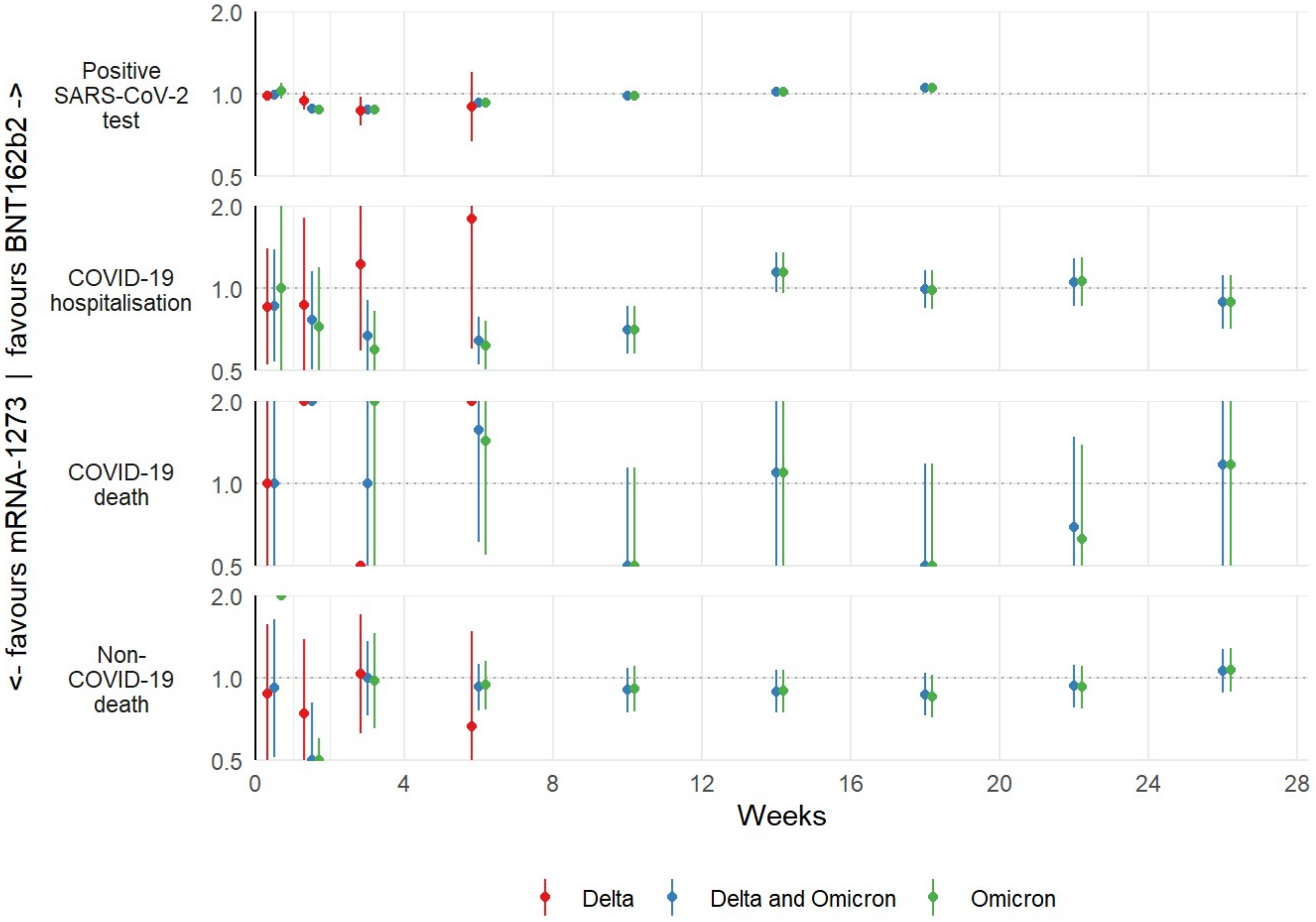

Table S5: 20-week Cox hazard ratios by variant era

Delta is defined as days up to 14 December 2021. Omicron is defined as day from 15 December 2021 onwards.

Time since boosting	Delta and Omicron	Delta	Omicron
Positive SARS-CoV-2 test			
1 - 7	0.99 (0.97 to 1.01)	0.98 (0.96 to 1.01)	1.03 (0.96 to 1.10)
8 - 14	0.88 (0.86 to 0.90)	0.95 (0.88 to 1.02)	0.88 (0.86 to 0.90)
15 - 28	0.88 (0.86 to 0.89)	0.86 (0.76 to 0.98)	0.87 (0.86 to 0.89)
29 - 56	0.93 (0.92 to 0.94)	0.90 (0.67 to 1.20)	0.93 (0.92 to 0.94)
57 - 84	0.98 (0.97 to 1.00)	NA (NA to NA)	0.99 (0.97 to 1.00)
85 - 112	1.01 (1.00 to 1.03)	NA (NA to NA)	1.01 (1.00 to 1.03)
113 - 140	1.05 (1.02 to 1.07)	NA (NA to NA)	1.05 (1.02 to 1.07)
COVID-19 hospitalisation			
1 - 7	0.86 (0.54 to 1.39)	0.86 (0.53 to 1.40)	1.00 (0.14 to 7.10)
8 - 14	0.76 (0.50 to 1.16)	0.87 (0.41 to 1.82)	0.72 (0.44 to 1.20)

Time since boosting	Delta and Omicron	Delta	Omicron
15 - 28	0.67 (0.50 to 0.91)	1.23 (0.59 to 2.56)	0.60 (0.43 to 0.83)
29 - 56	0.64 (0.53 to 0.79)	1.80 (0.60 to 5.37)	0.62 (0.50 to 0.76)
57 - 84	0.71 (0.58 to 0.87)	NA (NA to NA)	0.71 (0.58 to 0.86)
85 - 112	1.15 (0.97 to 1.35)	NA (NA to NA)	1.14 (0.96 to 1.35)
113 - 140	0.99 (0.85 to 1.17)	NA (NA to NA)	0.99 (0.84 to 1.16)
141 - 168	1.06 (0.86 to 1.29)	NA (NA to NA)	1.06 (0.87 to 1.30)
169 - 196	0.89 (0.71 to 1.12)	NA (NA to NA)	0.89 (0.71 to 1.12)
COVID-19 death			
1 - 7	1.00 (0.06 to 15.99)	1.00 (0.06 to 15.99)	NA (NA to NA)
8 - 14	Inf (Inf to Inf)	Inf (Inf to Inf)	NA (NA to NA)
15 - 28	1.00 (0.14 to 7.10)	0.0000 (0.0000 to 0.0000)	2.00 (0.18 to 22.06)
29 - 56	1.57 (0.61 to 4.05)	Inf (Inf to Inf)	1.43 (0.54 to 3.75)
57 - 84	0.36 (0.12 to 1.14)	NA (NA to NA)	0.36 (0.12 to 1.14)
85 - 112	1.09 (0.48 to 2.47)	NA (NA to NA)	1.09 (0.48 to 2.47)
113 - 140	0.42 (0.15 to 1.18)	NA (NA to NA)	0.42 (0.15 to 1.18)
141 - 168	0.69 (0.32 to 1.48)	NA (NA to NA)	0.63 (0.28 to 1.38)
169 - 196	1.17 (0.39 to 3.48)	NA (NA to NA)	1.17 (0.39 to 3.47)
Non-COVID-19 death			
1 - 7	0.92 (0.51 to 1.63)	0.88 (0.49 to 1.57)	Inf (Inf to Inf)
8 - 14	0.49 (0.29 to 0.81)	0.74 (0.39 to 1.38)	0.23 (0.09 to 0.60)
15 - 28	1.00 (0.73 to 1.37)	1.03 (0.63 to 1.71)	0.98 (0.65 to 1.46)
29 - 56	0.93 (0.76 to 1.13)	0.67 (0.30 to 1.48)	0.94 (0.77 to 1.16)
57 - 84	0.90 (0.75 to 1.09)	NA (NA to NA)	0.91 (0.75 to 1.11)
85 - 112	0.89 (0.74 to 1.07)	NA (NA to NA)	0.89 (0.75 to 1.07)
113 - 140	0.87 (0.73 to 1.04)	NA (NA to NA)	0.86 (0.72 to 1.03)
141 - 168	0.93 (0.78 to 1.12)	NA (NA to NA)	0.93 (0.77 to 1.11)
169 - 196	1.06 (0.88 to 1.27)	NA (NA to NA)	1.07 (0.89 to 1.29)

Estimands

The target estimand(s) is defined as the risk difference at 20 weeks of each outcome between those receiving a Moderna booster dose compared with a Pfizer booster dose, in those eligible and matched according to the stated eligibility and matching criteria. We also report the corresponding risk ratios and period-specific hazard ratios. None of the people in either group were excluded due to non-compliance; everybody received their assigned exposure of booster vaccination, a point-treatment with no possibility for discontinuation once administered. The per-protocol effect therefore coincides with the intention-to-treat effect.

JCVI priority groups

The Joint Committee on Vaccine and Immunisation (JCVI) priority groups were defined as follows:

Priority group	Description
1	Residents in a care home for older adults<br>Staff working in care homes for older adults
2	All those 80 years of age and over<br>Frontline health and social care workers
3	All those 75-79 years of age
4	All those 70-74 years of age<br>Individuals aged 16-69 in a high risk group
5	All those 65-69 years of age
6	Adults aged 16-64 years in an at-risk group
7	All those 60-64 years of age
8	All those 55-59 years of age
9	All those 50-54 years of age
10a	All those 40-49 years of age
10b	All those 18-39 years of age

See original priority groups here: https://assets.publishing.service.gov.uk/government/uploads/system/uploads/attachment_data/file/1007737/Greenbook_chapter_14a_30July2021.pdf#page=15 See revised priority groups here: https://www.england.nhs.uk/coronavirus/wp-content/uploads/sites/52/2021/07/C1327-covid-19-vaccination-autumn-winter-phase-3-planning.pdf

Note that we excluded care home residents and health care workers in our analysis, so members of JCVI group 1 are not included and JCVI group 2 includes only those aged 80

and over. The original priority group list has 9 groups, with a 10th group implicitly defined as “everybody else”. Here we explicitly define this group, and split into two (10a and 10b) because of the earlier booster eligibility in the 40-49 group from 15 November 2021 onwards. (https://www.gov.uk/government/news/jcvi-issues-advice-on-covid-19-booster-vaccines-for-those-aged-40-to-49-and-second-doses-for-16-to-17-year-olds)

Codelists and variable definitions

All codelists can be found at `https://codelists.opensafely.org/codelist/<ID>`, substituting `<ID>` for the codelist identifier in the table below.

Further details of how these codes are combined are available in the project study definition, and subsequent analysis R scripts.

Variable	Data source	Notes	Values	Codelist identifier
**Demographics**				
Sex	Primary care record		Male; Female	
Age	Primary care record		>=18	
Ethnicity	Primary care record; SUS-APCS	Taken from primary care record where known, and supplemented by SUS-APCS data where unknown	Black; Mixed; South Asian; White; Other	opensafely/ethnicity/2020-04-27/
NHS region	Primary care record		East; London; Midlands; North East and Yorkshire; North West; South East; South West	
Sustainability and Transformation Partnership	Primary care record	GPs are nested within STPs, so is taken from practice, not patient address.	One of 36 STPs	
English Index of Multiple Deprivation	Primary care record	Derived from Middle Layer Super Output Area (LSOA) of patient's address and using 2019 LSOA rankings	Grouped into 5 categories by quintile of IMD rank	
Care home residency	Primary care record; Care and Quality Commission care home address data	Patients were considered to be care-home residents if they met any one of three criteria used to identify care homes. These criteria are based on either patient address, household age and size, or coded clinical events. See https://wellcomeopenresearch.org/articles/6-90/v1 for more details on how these flags are derived.	0/1	primis-covid19-vacc-uptake/longres/v1 primis-covid19-vacc-uptake/carehome/v1 primis-covid19-vacc-uptake/nursehome/v1 primis-covid19-vacc-uptake/domcare/v1
Health and social care worker status	NIMS	Vaccinees were asked if they were a health care worker or carer at the time of vaccination.	0/1	
**Clinical**				
Severe obesity	Primary care record	BMI >= 40, based on most recent weight measurement, or clinically coded severe obesity	0/1	primis-covid19-vacc-uptake/sev_obesity/v1.2 primis-covid19-vacc-uptake/bmi_stage/v1.2 primis-covid19-vacc-uptake/bmi/v1
Asthma	Primary care record	Based on PRIMIS specification	0/1	primis-covid19-vacc-uptake/ast/v1 primis-covid19-vacc-uptake/astadm/v1 primis-covid19-vacc-uptake/astrx/v1
Chronic respiratory disease	Primary care record	Based on PRIMIS specification	0/1	primis-covid19-vacc-uptake/resp_cov/v1
Chronic heart disease	Primary care record	Based on PRIMIS specification	0/1	primis-covid19-vacc-uptake/chd_cov/v1.2.1
Chronic kidney disease	Primary care record	Based on PRIMIS specification	0/1	primis-covid19-vacc-uptake/ckd_cov/v1.2.1 primis-covid19-vacc-uptake/ckd15/v1 primis-covid19-vacc-uptake/ckd35/v1
Chronic liver disease	Primary care record	Based on PRIMIS specification	0/1	primis-covid19-vacc-uptake/cld/v1
Diabetes	Primary care record	Based on PRIMIS specification	0/1	primis-covid19-vacc-uptake/dmres/v1 primis-covid19-vacc-uptake/diab/v1

Chronic neurological disease	Primary care record	Based on PRIMIS specification	0/1	primis-covid19-vacc-uptake/cns_cov/v1
Immunosuppressed	Primary care record	Based on PRIMIS specification	0/1	primis-covid19-vacc-uptake/immdx_cov/v1 primis-covid19-vacc-uptake/immdx/v1
Immunosuppressive medications	Primary care record	Based on PRIMIS specification, within the last 6 months	0/1	primis-covid19-vacc-uptake/immdx/v1
Asplenia	Primary care record	Based on PRIMIS specification	0/1	primis-covid19-vacc-uptake/spln_cov/v1/
Cancer, non-haematological	Primary care record	Within the last 3 years	0/1	opensafely/cancer-excluding-lung-and-haematological-snomed/2020-04-15/ opensafely/lung-cancer-snomed/2020-04-15/ opensafely/chemotherapy-or-radiotherapy-snomed/2020-04-15/
Cancer, haematological	Primary care record	Within the last 3 years	0/1	opensafely/haematological-cancer-snomed/2020-04-15/
Solid organ transplantation	Primary care record		0/1	opensafely/solid-organ-transplantation-snomed/2020-04-10/
HIV/AIDS	Primary care record		0/1	nhsd/hiv-aids-snomed/68ca529e/
Severe mental illness	Primary care record	Based on PRIMIS specification	0/1	primis-covid19-vacc-uptake/sev_mental/v1 primis-covid19-vacc-uptake/smhres/v1
Learning disabilities	Primary care record	Based on PRIMIS specification	0/1	primis-covid19-vacc-uptake/learndis/v1
End-of-life care	Primary care record	Based on codes indicating participation in end-of-life or palliative care pathways, or Midalozam prescriptions	0/1	nhsd-primary-care-domain-refsets/palcare_cod/5fce98cf opensafely/midazolam-end-of-life/4c1b3c89
Housebound	Primary care record		0/1	opensafely/housebound/5bc77310 primis-covid19-vacc-uptake/carehome/v1 opensafely/no-longer-housebound/29a88ca6
Clinically extremely vulnerable	Primary care record	Based on PRIMIS specification. This is also known as the Shielding list	0/1	primis-covid19-vacc-uptake/shield/v1 primis-covid19-vacc-uptake/nonshield/v1
Clinically at-risk	Primary care record	immunosuppressed; asplenia; chronic heart disease; chronic kidney disease; chronic liver diease; diabetes; chronic respiratory disease; asthma; chronic neurological disease; learning disabilities; severe mental illness	0/1	
Comorbidity count	Primary care record	Count of: severe obesity; chronic heart disease; chronic kidney disease; chronic liver diease; diabetes; chronic respiratory disease OR asthma; chronic neurological disease	>=0	
In-hospital status, planned	SUS-APCS	With admission method in ["11", "12", "13", "81"]	Date	
In-hospital status, unplanned	SUS-APCS	With admission method in ["21", "22", "23", "24", "25", "2A", "2B", "2C", "2D", "28"]	Date	
**Vaccination**				
Vaccination status	NIMS / Primary care record	Vaccination details are recorded in the National Immunisation Management Service (NIMS) and transmitted to the patient's GP record within days. Vaccination status can be identified from by the vaccine product name. Vaccine cardinality (i.e., first dose, second etc) is identified by vaccine dates, rather than explicit clinical coding. There is no way to unambiguously distinguish	Date + BNT162b2; ChAdOx1-S; mRNA-1273	

		“third primary dose” and “first booster dose” as the indication for vaccination is not recorded in the vaccine record. This must be inferred by vaccine date and clinical characteristics of the patient.		
**COVID-19 events**				
Probable COVID-19	Primary care record	Exact dates of COVID-19 infection / disease in priamry care records are unreliable. This variable is only used to determine historical infection prior to the study start date. Important due to low availability of testing early in the pandemic	Date	opensafely/covid-identification-in-primary-care-probable-covid-positive-test/2020-07-16/ opensafely/covid-identification-in-primary-care-probable-covid-clinical-code/2020-07-16/ opensafely/covid-identification-in-primary-care-probable-covid-sequelae/2020-07-16/
SARS-CoV-2 positive test	SGSS	Any positive SARS-CoV-2 test, whether PCR or LFT. Swab date is used as the event date, not the date that the result was recorded.	Date	
Count of prior tests in previous x days	SGSS	The number of SARS-CoV-2 tests, whether PCR or LFT, recorded x days prior to booster vaccination. Swab date is used as the event date, not the date that the result was recorded.	>=0	
COVID-19 A&E attendances	SUS-ECDS	Any registered attendance to A&E with COVID-19 ICD10 codes mentioned anywhere in the “discharge diagnosis” field (not just the primary diagnosis)	Date	opensafely/covid-identification/2020-06-03/
COVID-19 Hospital admissions	SUS-APCS	Any (completed) hospital episode with COVID-19 ICD10 codes mentioned anywhere in the diagnosis field (not just the primary diagnosis), and with admission method in ["21", "22", "23", "24", "25", "2A", "2B", "2C", "2D", "28"]	Date	opensafely/covid-identification/2020-06-03/
COVID-19 death	Death register (ONS)	Deaths with COVID-19 ICD10 diagnosis codes mentioned anywhere on the death certificate (not just underlying cause)	Date	opensafely/covid-identification/2020-06-03/
Prior COVID-19 event within x days	(derived)	Any SARS-CoV-2 positive test, COVID-19 hospital admissions, or COVID-19 A&E attendances occurring within x days of booster vaccination. Note, we do not used “probable covid in primary care” to derive this variable as the entry date for this code may not reflect the date of the infection episode.	0/1	
Prior COVID-19 event ever	(derived)	Any SARS-CoV-2 positive test, COVID-19 hospital admissions, COVID-19 A&E attendances or Probable COVID-19 as recorded in primary care, occurring before booster vaccination.	0/1	
Death	Death register (ONS)	Any death	Date	
